# Quantitative proteomics reveals the dynamic proteome landscape of zebrafish embryos during the maternal-to-zygotic transition

**DOI:** 10.1016/j.isci.2024.109944

**Published:** 2024-05-08

**Authors:** Fei Fang, Daoyang Chen, Abdul Rehman Basharat, William Poulos, Qianyi Wang, Jose B. Cibelli, Xiaowen Liu, Liangliang Sun

**Affiliations:** 1 Department of Chemistry, Michigan State University, 578 S Shaw Lane, East Lansing, MI 48824, USA; 2 Department of BioHealth Informatics, Indiana University-Purdue University Indianapolis, Indianapolis, IN 46202, USA; 3 Department of Animal Science, Michigan State University, East Lansing, MI 48824, USA; 4 Department of Large Animal Clinical Sciences, Michigan State University, East Lansing, MI 48824, USA; 5 Deming Department of Medicine, School of Medicine, Tulane University, 1441 Canal Street, New Orleans, LA 70112, USA

**Keywords:** Developmental biology, Proteomics

## Abstract

Maternal-to-zygotic transition (MZT) is central to early embryogenesis. However, its underlying molecular mechanisms are still not well described. Here, we revealed the expression dynamics of 5,000 proteins across four stages of zebrafish embryos during MZT, representing one of the most systematic surveys of proteome landscape of the zebrafish embryos during MZT. Nearly 700 proteins were differentially expressed and were divided into six clusters according to their expression patterns. The proteome expression profiles accurately reflect the main events that happen during the MZT, i.e., zygotic genome activation (ZGA), clearance of maternal mRNAs, and initiation of cellular differentiation and organogenesis. MZT is modulated by many proteins at multiple levels in a collaborative fashion, i.e., transcription factors, histones, histone-modifying enzymes, RNA helicases, and P-body proteins. Significant discrepancies were discovered between zebrafish proteome and transcriptome profiles during the MZT. The proteome dynamics database will be a valuable resource for bettering our understanding of MZT.

## Highlights

• Expression dynamics of 5,000 proteins during the zebrafish MZT is revealed
• Proteome expression profiles reflect the main events that happen during the MZT
• Embryonic MZT is modulated at multiple levels in a collaborative fashion
• Obvious discrepancies exist between embryonic proteome and transcriptome profiles

## Graphical abstract

**Figure:**
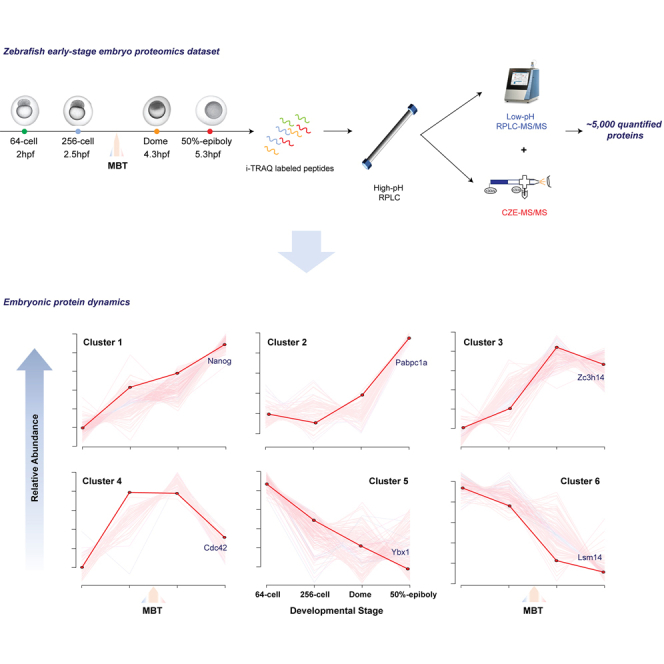

## Introduction

In many organisms, early embryonic development is modulated by maternally supplied RNAs and proteins that have been stockpiled in the oocyte. Following the clearance of a subset of these maternal products, the zygotic transcription is initiated, namely zygotic genome activation (ZGA).^1^^,^^2^^,^^3^^,^^4^ Clearance of maternal products (i.e., RNAs) and initiation of ZGA are two main interrelated processes during the maternal-to-zygotic transition (MZT), which is vital for embryogenesis. Tremendous amounts of efforts have been made to better our understanding of the underlying mechanisms of MZT, including conventional biochemical approaches or omics strategies (i.e., transcriptomics and proteomics).^5^^,^^6^^,^^7^^,^^8^^,^^9^^,^^10^^,^^11^^,^^12^^,^^13^^,^^14^^,^^15^^,^^16^^,^^17^^,^^18^^,^^19^^,^^20^^,^^21^ For example, it has been discovered that specific RNA binding proteins, small non-coding RNAs, and RNA modifications contribute to the clearance of maternal RNAs.^5^^,^^6^^,^^7^ Regarding the initiation of ZGA, transcriptional repressors (e.g., histone), transcriptional activators (i.e., transcription factors, TFs), and chromatin accessibility (i.e., overall genome becoming more compact and local genome accessibility increasing due to histone post-translational modifications (PTMs)) are critical.^11^^,^^22^^,^^23^^,^^24^ Mass spectrometry (MS)-based quantitative proteomics studies of early-stage embryos will provide a bird’s-eye view of the proteome dynamics during MZT and offer new insights into the controlling mechanisms of maternal RNA clearance and ZGA initiation.

Zebrafish (*Danio rerio*) is a widely used model organism for studying early vertebrate embryogenesis (i.e., MZT). There are two waves of ZGA in zebrafish,^25^ a minor wave during the cleavage period corresponding to hundreds of protein-coding genes^8^ and a major wave after midblastula transition (MBT) corresponding to thousands of protein-coding genes.^9^ For zebrafish, MBT is about 3 h postfertilization (hpf) at 28.5°C. Zebrafish has many valuable features. First, zebrafish and humans have good genetic similarities, and 70% of human genes have at least one zebrafish orthologue.^26^ Second, as a rapidly developing species, zebrafish begin embryonic development with a transcriptionally silent genome, complete the MBT, and enter gastrulation within only a few hours. Third, the TFs, which specify the on-and-off states of genes, are highly translated in the zebrafish embryos before the MBT,^11^ making them more amenable to being detected. Fourth, genetic manipulations such as transgenesis and morpholino knock-down can be implemented to determine specific proteins’ roles in developing embryos.^27^ Fifth, zebrafish embryos are transparent, which makes it easy to track embryonic development.

Tremendous amounts of studies have been done to study functions of specific proteins in modulating zebrafish MZT^7^^,^^11^^,^^22^^,^^23^^,^^28^^,^^29^ or to provide transcriptome dynamics profiling of zebrafish embryos during MZT.^12^^,^^13^^,^^25^^,^^30^^,^^31^ MS-based proteomics measurements of zebrafish embryos have also been done to achieve a global view of proteome dynamics during early embryogenesis.^17^^,^^18^^,^^19^^,^^20^^,^^21^ However, some technical challenges exist for deep proteomics of zebrafish early-stage embryos (i.e., during MZT) due to the extremely high abundant yolk proteins in the embryos, which interferes with the identification of low-abundance proteins.^32^ Deyolking has been employed for proteomic characterization of zebrafish embryos to boost proteome coverage, yielding 2–3 times more identified proteins than non-deyolked embryos.^33^^,^^34^ Deyolking was also used in one most recent label-free quantitative proteomics study of zebrafish embryos across 10 developmental stages with the identification of 5961 proteins in total and about 2000 proteins at each stage.^20^ However, deyolking could lead to the loss of development-related proteins.^35^ Alternatively, multidimensional liquid-phase separations of peptides, i.e., two-dimensional liquid chromatography (2D-LC) and LC-capillary zone electrophoresis (CZE), before MS have been successfully utilized for deep proteomics profiling of complex samples because of the improvement of peptide separation capacity.^36^^,^^37^^,^^38^

In this work, we aim to investigate the proteome landscape of zebrafish embryos without deyolking across four embryonic stages (64-cell, 256-cell, dome, and 50% epiboly stages) during the MZT using both 2D-LC-tandem MS (MS/MS) and LC-CZE-MS/MS. We used reversed-phase LC (RPLC)-CZE-MS/MS and 2D-RPLC-MS/MS to boost the number of peptide and protein identifications because CZE-MS/MS and RPLC-MS/MS are complementary for peptide and protein identifications.^39^ Without embryo deyolking, we identified and quantified nearly 5,000 proteins from the zebrafish embryos across the four stages. The proteome dataset unveils the expression of protein clusters associated with critical events happening during the first 6 h of the zebrafish embryos’ life, i.e., clearance of maternal RNAs, ZGA, initiation of cellular differentiation and organogenesis. We further extended our proteome dataset with published transcriptome dataset for a better understanding of the relationship between transcript and protein during the MZT.

## Results and discussion

### Quantification of nearly 5000 proteins from zebrafish early-stage embryos using 2D-RPLC-MS/MS and RPLC-CZE-MS/MS

**Figure 1:**
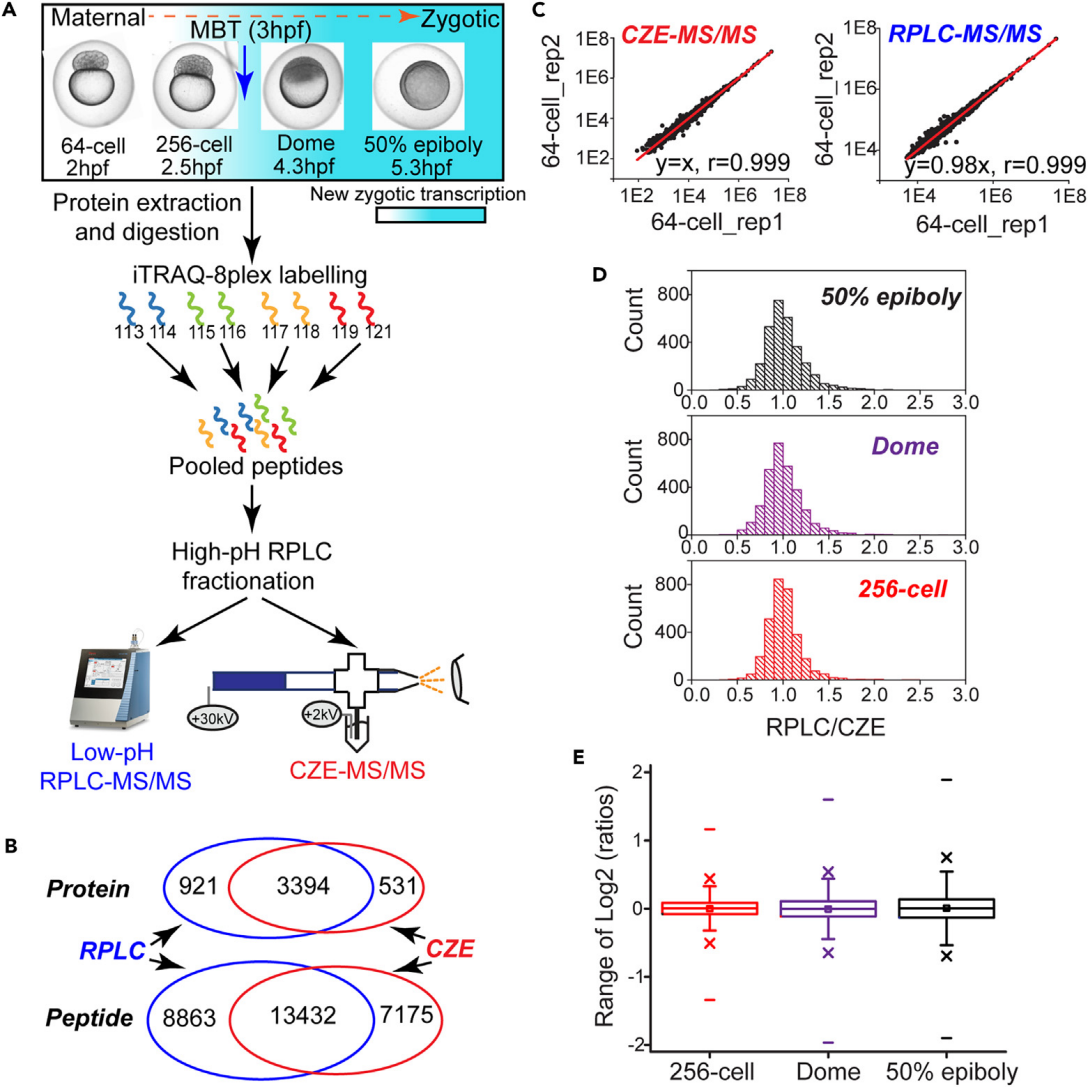
Summary of the quantified proteins from zebrafish embryos by CZE-MS/MS and RPLC-MS/MS (A) Workflow of the iTRAQ-based quantitative experiment. Proteins were extracted from the embryos at four different stages in biological duplicate and digested. The peptides labeled with 8 channels of iTRAQ reagent were mixed and fractionated using high-pH RPLC, followed by low-pH RPLC-MS/MS and CZE-MS/MS analysis. (B) Overlaps of quantified proteins and peptides by RPLC-MS/MS and CZE-MS/MS. (C) Examples of reporter ion intensity correlations between biological replicates at the 64-cell stage from CZE-MS/MS and RPLC-MS/MS analyses. (D) Normalized reporter ion intensity ratio (RPLC/CZE) distributions of overlapped proteins at the 256-cell, Dome, and 50% epiboly stages. The reporter ion intensity of proteins from CZE or RPLC were averaged across biological duplicates at each stage, and the reporter ion intensity from the 256-cell, Dome, and 50% epiboly stages were divided by that at the 64-cell stage for normalization. The obtained intensity ratios from RPLC were divided by that from CZE at the same stage and the produced ratios were used in the figure. (E) Boxplots of log_2_(reporter ion intensity ratios) at the 256-cell, Dome, and 50% epiboly stages from the combined CZE and RPLC data. The reporter ion intensity of those three stages were normalized to the 64-cell stage for CZE and RPLC separately. For the overlapped proteins, the ratios from CZE and RPLC were averaged for each stage. Otherwise, the CZE or RPLC data were used. In total, 4846 proteins were quantified and used in the figure.

To achieve a bird’s-eye view of the proteome dynamics during MZT, we performed a deep proteome profiling of zebrafish embryos across four developmental stages, including 64-cell (2 hpf), 256-cell (2.5 hpf), dome (4.3 hpf), and 50%-epiboly (5.3 hpf) stages, Figure 1A. From the 64-cell stage to the 50% epiboly stage, the zebrafish embryos gradually undergo the transition from maternal to zygotic. At each time point, two biological replicates of zebrafish embryos (pools of 15 embryos) were collected (eight samples in total), lysed without deyolking, and digested with trypsin. The produced peptides were labeled by the iTRAQ-8plex (isobaric tag for relative and absolute quantitation) chemistry. Then, the pooled peptides were separated by high-pH RPLC into 50 fractions, followed by low-pH RPLC-MS/MS and CZE-MS/MS analyses, respectively.

4,413 and 4,315 proteins were identified and quantified by 2D-RPLC-MS/MS, resulting from 22,295 peptides. Meanwhile, CZE-MS/MS identified 20,607 peptides, corresponding to the identification and quantification of 4,008 and 3,925 proteins, respectively (Table S1). As shown in Figure 1B, CZE-MS/MS and RPLC-MS/MS offered good orthogonality for peptide and protein identifications (IDs), producing a 32% increase in peptide IDs and a 12% improvement in protein number after the combination of those two techniques compared to RPLC-MS/MS alone. In comparison with the RPLC-MS/MS, the CZE-MS/MS could identify not only the peptides with higher molecular weight (Figure S1A) but also those containing more hydrophobic amino acids (Figures S1B and S1C), indicating that the difference might be attributed to the improved recovery of large and hydrophobic peptides by CZE compared to RPLC. Furthermore, the combination of CZE-MS/MS and RPLC-MS/MS boosted the average sequence coverage of proteins from 11% to 15%, Figure S1D. The above results underline the improved proteome coverage facilitated by the high complementarity between CZE and RPLC-based platforms.

Both RPLC-MS/MS and CZE-MS/MS platforms produced confident protein quantification data. First, iTRAQ reporter ion intensities between biological replicates are consistent at all four stages and have high linear correlations with the Pearson’s r better than 0.99 in all cases and the slope close to 1 (1.1 ± 0.1). Figure 1C shows the examples of intensity correlations at the 64-cell stage for CZE-MS/MS and RPLC-MS/MS. Second, CZE-MS/MS and RPLC-MS/MS data agree well with each other regarding the normalized protein expression relative to the 64-cell stage, Figure 1D. The normalized protein expression of RPLC-MS/MS at the 256-cell, dome, and 50% epiboly stages was divided by that of CZE-MS/MS and the ratio distributions center at 1 with standard deviations of about 0.2. Third, 4846 proteins were quantified after combining the CZE- and RPLC-MS/MS data. For the overlapped proteins, the normalized protein expression relative to the 64-cell stage from CZE and RPLC were averaged at each stage. The distributions of normalized protein expression become wider gradually from the 256-cell stage to 50% epiboly stage, Figure 1E, suggesting the protein expression changes are more significant as the embryos grow, which agrees well with the embryonic phenotype changes. Lastly, we revealed significant upregulation of Nanog and Ctcf, substantial downregulation of Ybx1, and relative consistent expression of Pou5f3 from the 64-cell (2 hpf) to 50% epiboly (5.3 hpf) stages in our proteomics study. Our quantitative proteomics data agree well with their western blot results reported in the literature.^40^^,^^41^^,^^42^^,^^43^

We compared our proteomics data with published Ribo-Seq (ribosome sequencing) data from the Giraldez group.^11^^,^^30^ Ribo-Seq offers a “snapshot” of all active ribosomes in cells at a specific time point, which helps us identify the proteins that are being actively translated in the cells and provide information about translation level of each protein by the ribosome occupancy.^44^^,^^45^ However, it cannot provide accurate information about protein abundance in cells, which is controlled by both translation rate and degradation rate. It also cannot provide information of protein PTMs. Quantitative proteomics directly measure proteins globally in cells, tissues, and biofluids, providing identification, quantification, and PTM information of proteins. The RNA-Seq data of zebrafish embryos at 2 hpf detected translation events and translation level of roughly 10,000 mRNAs.^11^^,^^12^^,^^30^ The data suggests that about 10,000 proteins can be translated in the early-stage zebrafish embryos, which agrees reasonably well with the proteomics data of *Xenopus* embryos in terms of the number of proteins.^46^ However, since some proteins in early-stage zebrafish embryos can be maternally deposited, independent of mRNA levels and translation, it is central to directly measure proteins to gain an accurate picture of the proteome of early-stage zebrafish embryos. We quantified nearly 5,000 proteins from the zebrafish embryos here. More efforts are needed to reduce the interference of high-abundance yolk proteins for better proteome coverage. According to the Ribo-Seq data, Nanog, Sox19b, and Pou5f1 are highly translated proteins at the 2 hpf, ranking 25, 101, and 185 out of 10,000, respectively.^11^ Interestingly, our proteomics data determined that Nanog and Pou5f1 (Pou5f3) are medium abundance proteins in zebrafish embryos at the 2 hpf, ranking 1,089 and 2,563 out of about 4,000 proteins, respectively, based on the reporter ion intensity of iTRAQ. We did not identify the Sox19b protein from the 2hpf embryos, implying its relatively low abundance. Some high abundant proteins based on the iTRAQ reporter ion intensity include yolk proteins (vtg1-vtg7, most abundant proteins) and Ybx1 (ranking 347). The results here highlight the discrepancy between rib-seq and proteomics regarding the determination of protein abundance in a biological system. According to the RNA-seq data from Lee et al.,^11^ about 97% of the identified proteins in our proteomics study have maternal contributions, and only less than 3% of them are strictly zygotic. The data clearly indicate that the biological processes in zebrafish embryos during the MZT are mainly controlled by maternal proteins. The data also suggests minor ZGA during the cleavage period because zygotic proteins were detectable at the 64-cell stage.

We further compared our proteomics data with one recent proteomics study of zebrafish embryos. Yan et al. studied zebrafish embryonic proteome using a label-free approach across 10 different stages from the 4-cell stage to 5 days post fertilization.^20^ With deyolking step, they identified 5,961 proteins in total and about 2000 proteins at each stage. In our study, 4,846 proteins were identified at each embryonic stage without deyolking using the iTRAQ-8plex chemistry. To make fair comparisons, we chose the data from two embryonic stages (the 256-cell and dome stages) that were included in both studies.

Our study identified 4,846 proteins from about 4,300 unique genes, about 140% more proteins than Yan’s study, which identified about 2,000 proteins from about 1,900 unique genes from embryos at the two stages. Interestingly, only about 60% of the genes identified from Yan’s study were covered by our study. The genes unique to our study and Yan’s work have drastically different biological process enrichment results, Figure S2. The drastic differences in protein identification may be due to several reasons. First, we worked on the embryos directly without deyolking and Yan et al. measured the embryos after deyolking. Second, we utilized both 2D-RPLC-MS/MS and RPLC-CZE-MS/MS for peptide identification. Yan et al. deployed RPLC-MS/MS for the measurement. Those two reasons could mainly contribute to the substantially higher protein identifications in our study compared to Yan’s work for the 256-cell and dome stages. Lastly, our work employed the iTRAQ labeling-based proteomics and Yan et al. utilized a label-free-based proteomics approach. The iTRAQ labeling affects the peptide separation and identification by LC-MS/MS substantially due to its significant impact on peptides’ size, hydrophobicity, and gas-phase fragmentation. This may be one important reason why only 60% of the genes from Yan’s study were covered by our study.

We further compared the protein quantification data between Yan’s work and our work for 256-cell and dome stages. About 1,135 proteins were quantified at the 256-cell and dome stages in both studies and the protein intensity ratios of the two stages correlate poorly between the two studies, Figure S3. It’s worth noting that if we only consider the overlapped differentially expressed proteins during the MZT from the two studies, the correlation is reasonable (Pearson’s r = 0.82), Figure S4. The phenomenon observed in Figure S3 could be due to the potential issues of iTRAQ and label-free-based quantitative proteomics. iTRAQ-based approach has the potential issue of protein abundance ratio compression due to co-fragmentation of peptides,^47^ while the label-free-based approach tends to have significant intensity variations across technical or biological replicates since each replicate needs to be measured by CZE/RPLC-MS/MS separately.

### Clusters of quantified proteins based on their expression patterns

To investigate the global temporal patterns of proteomic changes, a time-series differential expression analysis was performed on our proteome dataset with maSigPro package.^48^ Among the total quantified 4846 proteins (Table S2), the proteins, whose expression level with absolute log_2_ fold change>0.2 between any two-time points, with an adjusted false discovery rate (FDR) corrected *p*-value<0.05, were determined as proteins with statistically significant abundance changes.

**Figure 2:**
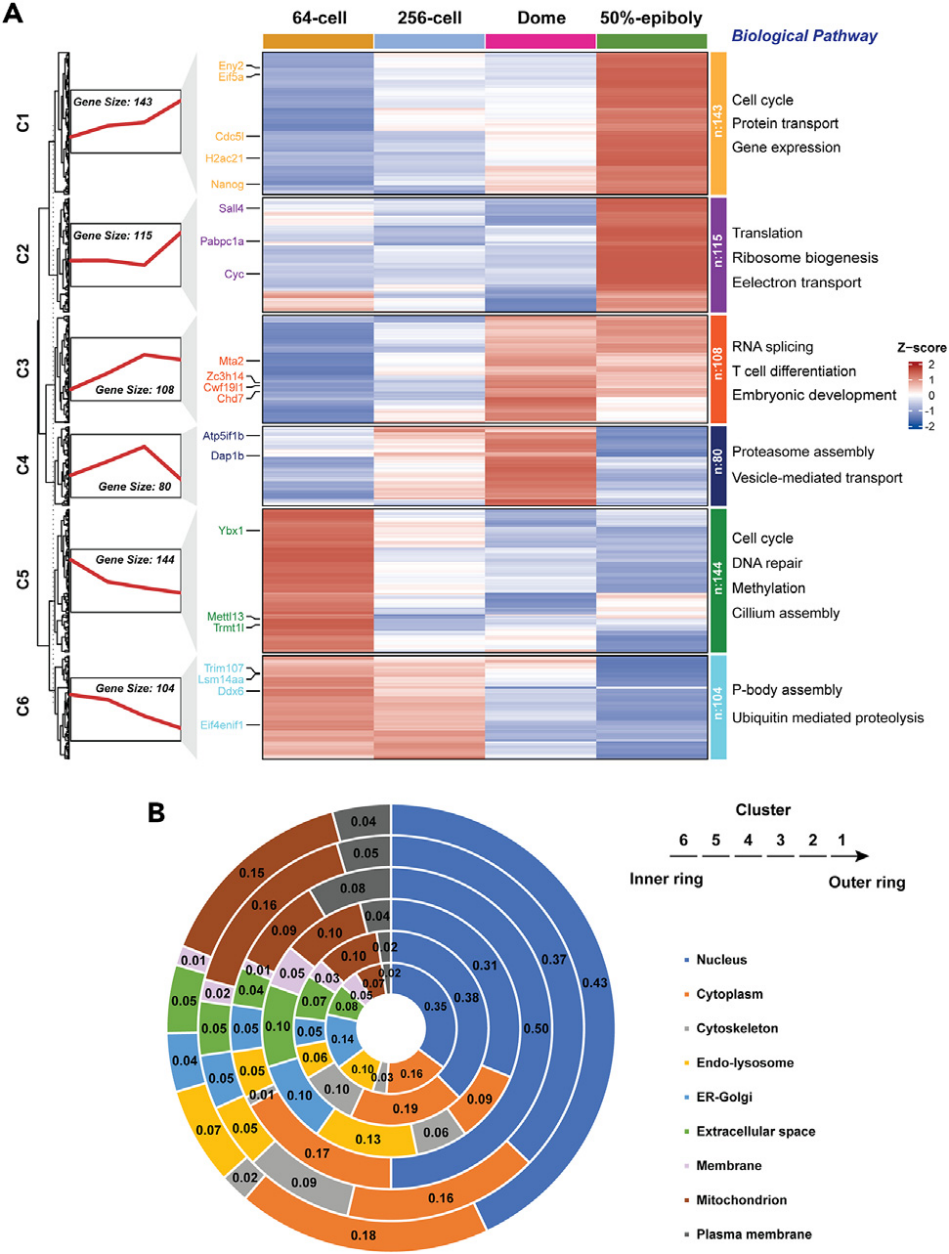
Cluster analysis of differentially expressed proteins quantified with RPLC-MS/MS and CZE-MS/MS methods (A) Heatmap in the middle showing distinct expression profiles of different clusters at each developmental stage. Fuzzy clustering of the expression data along zebrafish embryo early development stages are shown at left. The functional enrichment data of the proteins in each cluster are shown at right (*p*-value<0.1). The smaller the *p*-value is, the higher enrichment of genes in the annotation categories is. (B) Cellular component distribution of the differentially expressed proteins in each cluster. DAVID Bioinformatics Resources (https://david.ncifcrf.gov/) was used for the functional enrichment and cellular component analyses.

In total, 694 proteins were found differentially expressed across the 64-cell, 256-cell, dome, and 50%-epiboly stages and 4152 proteins have no statistically significant abundance changes across the four stages. The data suggests that the majority of the proteome is consistent during this period of development. Those differentially expressed proteins were clustered into six clusters according to their expression patterns using the ClusterGVis package,^49^ Figure 2A and Table S3. A specific website containing the complete quantitative proteomics dataset is now available to the scientific community (https://www.toppic.org/software/zebrafishdb/index.html).

Cluster 1 contains 143 proteins, whose expression level continuously increases from the 64-cell to 50%-epiboly stages. The proteins in this cluster are most likely from maternal contribution. To confirm this point, we compared our proteomics data with the RNA-seq datasets by Lee et al. and Bhat et al.^11^^,^^12^ We discovered that nearly 80% of cluster 1 proteins are from maternal mRNAs (e.g., Nanog, Ctcf, Cdc5l, Hells, and Ddx39aa) and only 5 proteins are from strictly zygotic contribution (e.g., Plagl2). One important model explaining MZT of zebrafish embryos is the accumulation of some transcriptional activators during the MZT (i.e., Nanog, Pou5f1 and SoxB1).^11^ Nanog in cluster 1 is a well-known TF accumulated during MZT for triggering the ZGA.^11^^,^^50^ Another accumulated TF in this cluster, Ctcf, is the only known vertebrate insulator protein and could silence somatic genes and upregulate pluripotency genes.^51^ It has been reported that Ctcf regulates the expression of thousands of genes during zebrafish early embryogenesis.^52^ Eny2 is a TF in this cluster and could play a role in regulation of transcription according to the data from *Drosophila* embryos.^53^ However, the possible function of Eny2 in transcription regulation in zebrafish embryos during MZT is largely unknown. Cdc5l, not only acts as a positive regulator of cell cycle G2/M progression, but also modulates pre-mRNA splicing,^54^ implying its potentially important roles during the MZT. Several helicases are also in this cluster, e.g., Hells (Lsh) and Ddx39aa. Hells can regulate transcription by collaborating with DNA methyltransferases in human cells.^55^ Ddx39a could modulate the expression of important genes related to the development of heart, skeletal muscle and lens of zebrafish embryos.^56^ The zygotic protein Plagl2 is critical for neurogenesis.^57^ The functional annotation data indicate significant enrichment (*p*-value≤ 0.05) of proteins related to, for example, cell cycle, translation, protein transport, proteasome, mitochondrion, ribonucleoprotein, and helicase for cluster 1 (Table S4).

Cluster 3 contains 108 proteins, and their expression reaches a plateau at the dome stage. According to the RNA-seq datasets by Lee et al. and Bhat et al.,^11^^,^^12^ 82% of proteins in this cluster originate from maternal mRNA (e.g., Pnn, Xpo4, Kpna2, Tnpo3, Ncl, Zc3h14, Mta2, and Chd7) and only 1 protein is strictly zygotic contribution (i.e., Fermt3a). Cluster 3 proteins could also play important roles in the MZT due to their dramatic accumulation, as evidenced by the significantly enriched biological processes, i.e., RNA splicing, nucleocytoplasmic transport, mRNA binding, and chromatin binding. Nucleocytoplasmic transport via importin modulates the ZGA in *Xenopus* embryos through controlling the timing of proteins entering the embryonic nuclei.^58^ Cluster 3 includes proteins related to the nucleocytoplasmic transport, e.g., Pnn (Pinin), Xpo4 (Exportin-4), Kpna2 (Importin subunit alpha), and Tnpo3 (Transportin 3). Pnn is crucial for regulating neural crest proliferation and differentiation in zebrafish embryos.^59^ Nucleolin (Ncl) involved in RNA binding modulates ribosomal RNA (rRNA) transcription and post-transcriptional regulation in zebrafish embryos.^60^ Zc3h14 is a polyadenosine [poly(A)] RNA-binding protein and controls the proper length of poly(A) tails.^61^ Zc3h14 also regulates pre-mRNA processing of some mRNA transcripts and modulates cellular energy levels.^62^ Several proteins related to chromatin binding are in this cluster, e.g., Mta2 and Chd7. Mta2 is one member of NuRD complex (nucleosome remodeling and histone deacetylation complex), which plays crucial roles in regulating DNA replication as a DNA replication factor during *Xenopus* early embryogenesis.^63^ Chd7 modulates left-right symmetry during zebrafish somitogenesis and is crucial for both early T cell development and thymus organogenesis.^64^^,^^65^ Chd7 co-localizes with Nanog, Oct4, and Sox2 in mouse embryonic stem cells.^66^

Cluster 2 has 115 proteins with elevated expression after the dome stage. According to the RNA-seq datasets by Lee et al. and Bhat et al.,^11^^,^^12^ 88 proteins in this cluster (77%) are from maternal contributions (e.g., Pabpc1a, Sall4, Ddx19b, Ddx3b, and Dhx38). Among them, 85% of the genes are strictly maternally provided or with average zygotic contribution less than 15.1% for transcription at 5.5 hpf, clearly demonstrating that the translation of maternal mRNAs can be delayed to even early gastrulation (i.e., 50% epiboly). Interestingly, only three proteins in this cluster are from strictly zygotic. The proteins in this cluster are highly enriched in translation, protein transport, RNA binding, and RNA helicase activity. The data suggests that some proteins in this cluster are related to the activities of MZT. One key activity of MZT is to clear the maternal mRNAs. In zebrafish, microRNAs (miRNAs) play critical roles for translational repression, deadenylation, and decay of maternal mRNAs during the MZT. For example, miRNA-430 is responsible for clearing about 40% of maternal mRNAs in zebrafish embryos during the MZT.^30^ It has been documented that miRNA-based poly(A) tail deadenylation and mRNA destabilization depends on the activity of poly(A)-binding protein (PABP).^67^ Pabpc1a in cluster 2 is a poly(A)-binding protein. The upregulation of Pabpc1a after the dome stage indicates its potential role in facilitating the decay of maternal mRNAs through the miRNA-based approach. Three helicases are in this cluster, Ddx19b, Ddx3b, and Dhx38. Ddx19b plays an important role in modulating the translation of newly synthesized mRNAs.^68^ Ddx3 is critical for neural crest development in *Xenopus* embryos,^69^ although the function of Ddx3b during zebrafish early embryogenesis is still unclear. Dhx38 contributes to the differentiation of hematopoietic stem cells, which can develop into various types of blood cells.^70^ One TF, Sall4, is also included in this cluster and it regulates genes that initiate hematopoiesis, contributing to the activation of the blood-specific program in zebrafish embryos.^71^ Sall4 also modulates the development of pectoral fin and taste epithelia in zebrafish.^72^^,^^73^ The data demonstrate that proteins in cluster 2 could play fundamental roles in early organogenesis.

Proteins in cluster 4 (80 proteins) show an interesting expression pattern with continuous accumulation before the dome stage and substantial decline after it. The expression profile implies that those proteins most likely function at the early stage of development. The protein expression declines due to the clearance of corresponding maternal mRNAs. Comparing the protein-level data with the RNA-seq datasets by Lee et al. and Bhat et al.,^11^^,^^12^ we determined that 62 proteins (74%) are from maternal mRNAs (e.g., Sept7b and Cdc42), 1 protein is from strictly zygotic contribution, and other proteins (17) are uncertain. The functional enrichment results indicate that proteins in this cluster are related to septin 7 (Sept7a and Sept7b), midbody (Cdc42, Sept7a, and Sept7b), and cilium (Ttll6, Sept7a, and Sept7b). Sept7b is critical for pronephric function and the establishment of left-right asymmetry in zebrafish embryogenesis.^74^ Midbody is crucial for embryonic cell division and pattern formation in zebrafish embryos,^75^ and Cdc42 contributes to the retinal development of zebrafish embryos.^76^ Cilium has many critical functions, including left-right asymmetry formation and sensory cell differentiation,^77^ and Ttll6 regulates cilia structure and motility.^78^

Cluster 5 has 144 proteins, and those proteins have a dramatic expression decline at the 256-cell stage right before the MBT. Out of the 144 proteins, 93 proteins are purely maternal (e.g., Ybx1, Dmap1, Rad50, and Ttf2) according to the RNA-seq data by Lee et al.^11^ Interestingly, 15 proteins in this cluster are from weak maternal contribution or strictly zygotic, suggesting the minor ZGA during the cleavage period. The data by Bhat et al.^12^ and Lee et al.^11^ agree in general. In zebrafish embryos, RNA binding proteins are crucial for clearance of maternal mRNAs and modulating the ZGA. For example, Ybx1 in this cluster stabilizes maternal mRNAs^29^ and represses global translation^79^ in early-stage zebrafish embryos. During the MZT, the Ybx1 facilitates the clearance of maternal mRNAs and the initiation of the ZGA. It has been documented that Ybx1 could regulate the processing of miRNA.^80^ We speculate that Ybx1 may modulate the maternal mRNA decay through regulating the miRNA-430, whose expression is controlled by TFs Nanog, Sox19b and Pou5f1 in zebrafish embryos.^11^ Several other proteins in this cluster, i.e., Dmap1 and Rad50, could also play fundamental roles as transcriptional repressors of the ZGA.^81^^,^^82^^,^^83^ Dmap1 activates Dnmt1 activity, leading to gene silencing in cells.^81^ Dmap1 contributes to transcriptional repression via targeting the replication foci together with Dnmt1 in cells.^82^ However, the role of Dmap1 in modulating the MZT of zebrafish embryos has not been probed. Our proteomics data suggests that the expression decline of Dmap1 in zebrafish embryos before the MBT might relate to the ZGA. Rad50 promotes DNA demethylation in cells and maintains cell pluripotency.^83^ However, the roles of Rad50 in the MZT of embryos have not been well studied. Our data documents the drastic decrease of its expression in zebrafish embryos before the MBT and suggests that it might facilitate the cellular differentiation that happens after the MBT. The data here fully demonstrate that proteins in this cluster could be fundamental for mediating the MZT. Proteins in this cluster are also involved in mRNA binding, export from nucleus, and posttranscriptional regulation, i.e., Zc3h11a.^84^ This cluster also contains proteins related to early organogenesis, for example, Ttf2 contributes to thyroid development.^85^ The functional enrichment data demonstrates that proteins in this cluster are significantly involved in DNA repair, ribosome biogenesis, mRNA binding and export, and methylation.

Proteins in cluster 6 (104 proteins) have similar expression profiles compared to cluster 5, but start the dramatic expression decline after the 256-cell stage (i.e., the dome stage). As expected, the majority of cluster 6 proteins (82%, 85 out of 104 proteins) are maternal (i.e., Lsm14a, Ddx6, Eif4enif1, and Paip2b).^11^^,^^12^ Only 3 proteins are strictly zygotic. The origin of the leftover 16 proteins is unknown. The proteins are highly enriched in cytoplasmic mRNA processing body (P-body) assembly, ubiquitin-mediated proteolysis, and negative regulation of translation. P-bodies are involved in mRNA storage for later use and mRNA decay in cells.^86^^,^^87^ In early-stage zebrafish embryos, maternal mRNAs may be stored and maintained in P-bodies and due to the need for mRNA clearance during the MZT, the P-body-related proteins are downregulated to release the stored maternal mRNAs for use and promote the clearance process. One member associated with P-bodies is Lsm14a, that functions as a maternal mRNA container and regulates protein expression in mouse oocytes.^88^ Our data suggests that its expression decline might relate to the maternal mRNA clearance during the MZT in zebrafish embryos. Ddx6 is another member of P-bodies foci, and it prevents cellular differentiation via degrading mRNAs of differentiation-inducing TFs.^89^ Lsm14 and Ddx6 directly interact and support the formation of mRNA silencing complexes and P-body assembly in cells.^90^ Our proteomics data indicate that Lsm14a and Ddx6 could be involved in mRNA silencing complexes for gene expression regulation (i.e., TFs) in zebrafish embryos during the MZT. Next, we investigated two proteins involved in the negative regulation of protein translation, Eif4enif1 and Paip2b. Paip2b functions as an inhibitor of protein translation.^91^ Eif4e binds to mRNAs and inhibit neurogenesis.^92^

We further studied the cellular components of the differentially expressed proteins in those six clusters, Figure 2B. About 30–50% of the proteins in each cluster are in the nucleus, suggesting the dramatic change of nucleus proteins during the MZT. About 10–20% of those proteins are in the cytoplasm and mitochondrion. We need to highlight that nearly 20% of proteins in clusters 1 and 2 are mitochondrion-related proteins, which is in accordance with the RNA-seq result^8^ and suggest that energy requirement becomes substantially higher with the progress of MZT.

The six different protein expression clusters revealed by our quantitative proteomics workflow accurately reflect the main events during the MZT, i.e., activation of zygotic genome, clearance of maternal mRNAs, and initiation of cellular differentiation and organogenesis. One previous quantitative proteomics study of *Xenopus* embryos also revealed six protein expression clusters, which are similar to the data of zebrafish embryos.^93^ Our proteomics dataset containing the expression dynamics of nearly 5,000 proteins provides us with a list of proteins that could play critical roles in collaboratively controlling the MZT. The dataset will undoubtedly be an invaluable resource for bettering our understanding of the MZT of vertebrate embryos.

### Expression dynamics of TFs during the MZT

**Figure 3:**
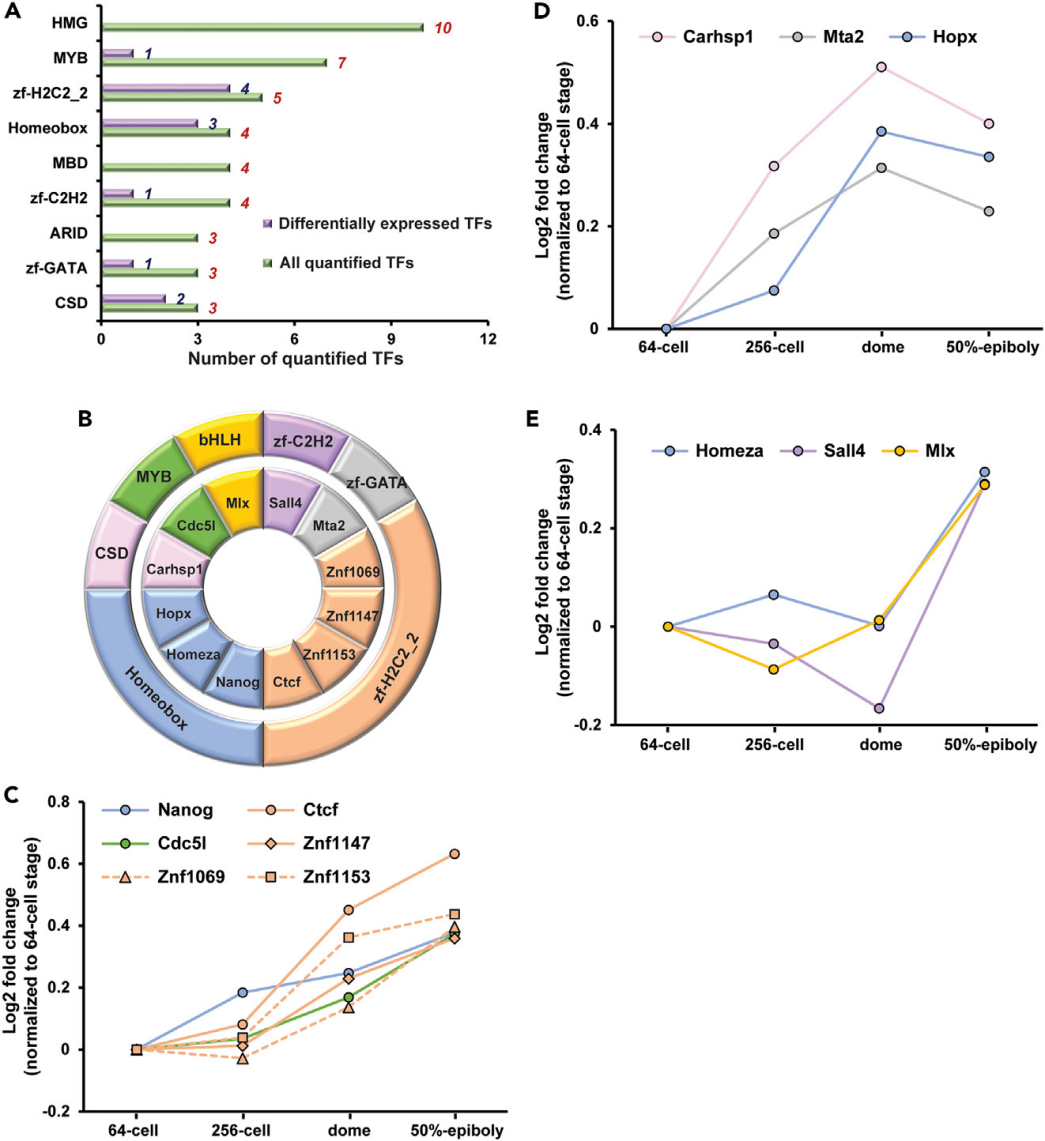
Expression level changes of the quantified transcription factors (TFs) (A) The quantified TFs and those with a significant change in expression level across four development stages were classified into families according to their DNA-binding domain composition. Families with fewer than three members were excluded. (B) DNA-binding domain distribution of the TFs upregulated during the MZT. (C and D) Expression profiles of TFs that show significant upregulation before the ZGA. (E) Expression profiles of TFs that have significant increase in abundance after the initiation of ZGA.

Given that the emergence of zygotic transcription in vertebrate embryos relies on TFs, defining the expression patterns of TFs during the MZT can better our understanding of the mechanisms that govern the MZT. After comparing our quantitative proteome dataset with the TF list predicted by Animal Transcription Factor DataBase (AnimalTFDB) 3.0,^94^ 73 annotated TFs were quantified with at least 2 peptides in our experiment. Those TFs are enriched in several families, such as the high-mobility group (HMG) TFs and zinc finger (ZF) proteins, Figure 3A and Table S5. TFs in the same family may have common structural traits and similar biological functions.

In total, 14 out of the 73 TFs have significant abundance changes across the four stages. Two TFs (Ybx1 and Pawrl) are significantly downregulated during the MZT. The expression of Pawrl starts to decline significantly after the dome stage. It is strictly maternal and functions as a transcriptional repressor in cells.^87^^,^^95^ In zebrafish embryos, it regulates TFs involved in eye development.^96^ Twelve TFs enriched in several families (e.g., homeobox and zinc finger-H2C2_2), Figure 3B, are showing significant upregulation during the MZT, Figures 3C–3E.

Nine TFs including Nanog exhibit significant accumulation before the dome stage, Figures 3C and 3D. They could relate to the regulation of MZT. Among them, Ctcf regulates the expression of thousands of genes during zebrafish early embryogenesis.^52^ Cdc5l could act as a positive regulator for cell proliferation.^54^ Hopx is a marker of hippocampal neural stem cells^97^ and modulates the cardiac development in zebrafish embryos.^98^ Mta2 functions as a DNA replication regulator in *Xenopus* early-stage embryos.^63^ Carhsp1 were reported to regulate transcript levels, maintaining the balance between pluripotency and differentiation.^99^ The expression profile data of those TFs provide some evidence of their potential biological functions in regulating DNA replication, transcription, or cellular differentiation in zebrafish embryos. Here, we also discovered three additional zinc finger TFs (Znf1147, Znf1069, and Znf1153) with significant accumulation of expression during the MZT. Their biological functions during the MZT have not been explored in the literature, but our proteomics data imply that those three zinc finger TFs might relate to the progression of MZT.

Three TFs show a boost in abundance at the 50% epiboly, Figure 3E. They most likely mediate temporal- or tissue-specific differentiation. Sall4, a zinc-finger TF, interacts with Oct4 and Nanog, forming protein complexes in mouse embryonic stem cells.^100^ Sall4 mediates the development of the blood-specific program, pectoral fin, and taste epithelia in zebrafish.^71^^,^^72^^,^^73^ It has been reported that Sall4 and Nanog form a regulatory circuit and co-occupy the enhancer regions in embryonic stem cells, demonstrating the important role of Sall4 in transcription regulation in collaboration with Nanog.^101^ Our quantitative proteomic data show that Nanog is continuously accumulated through the studied embryonic period and the expression of Sall4 is relatively consistent until the boost after the dome stage, implying diverse functions of Sall4 during the zebrafish early embryogenesis. Homeza is critical for normal neurogenesis in *Xenopus* emrbyos.^102^ Mlx contributes to myogenesis and muscle regeneration in mice.^103^ However, the functions of Homeza and Mlx in zebrafish embryos during the MZT have not been well documented in the literature.

### Expression dynamics of important proteins beyond TFs during the MZT

Apart from TFs, we also explored the expression patterns of other important proteins, including (1) histones and their modifying enzymes, (2) ribosomes, (3) RNA helicases, (4) glycolysis-related enzymes, as well as (5) P-bodies and protein degradation-related proteins.

#### Histones and their modifying enzymes

**Figure 4:**
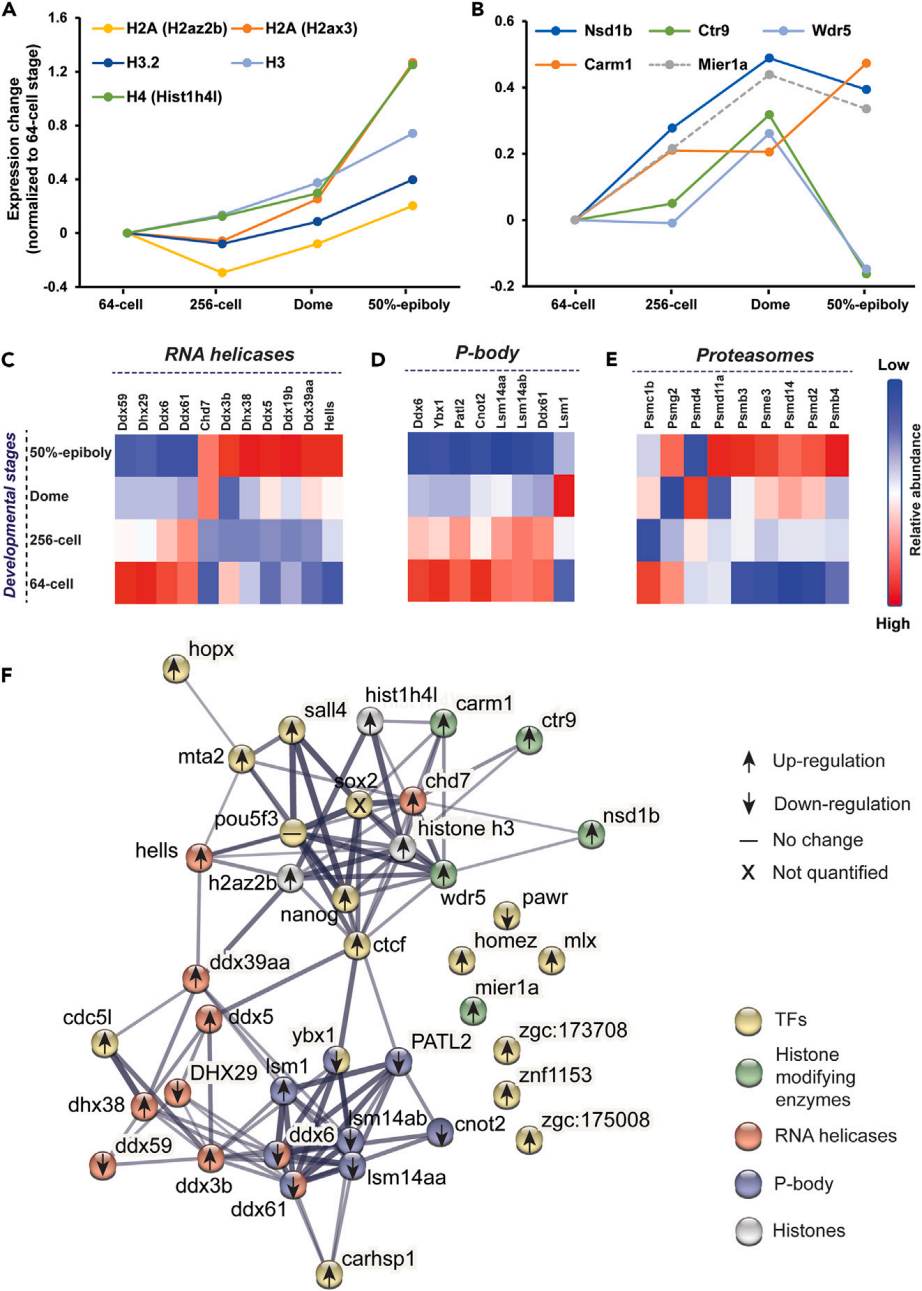
Expression patterns of potential regulators beyond TFs show significant abundance changes during the MZT Expression patterns of (A) histones, (B) histone-modifying enzymes, (C) RNA helicases, (D) P-body proteins, and (E) proteasomes. (F) Protein-protein interaction analysis of the differentially expressed TFs, histone-modifying enzymes, RNA helicases, and P-body proteins. The STRING (https://string-db.org/)^105^ was used for the analysis. Network type is full STRING network, and the edges indicate both physical and functional associations of proteins. The network edge suggests the confidence of protein-protein connection and the line thickness indicates the strength of data support with thicker lines as more confident connections. The minimum required interaction score is medium confidence (0.4). Others are default settings.

Histones assemble into histone octamers on DNA to form nucleosomes, the basic building blocks of chromatin. Most of the histone variants quantified have no substantial change in abundance before the MBT (∼1000-cell stage), Figure 4A. Our proteomics data agrees well with the western blot data for those histones before the major wave of ZGA.^23^ The rapid cell cleavage before the ZGA and relative consistent overall histone abundance lead to progressive dilution of histone repression on the genome activation.^104^ The dilution of histone on chromatin coincides with the accumulation of TFs, assuring the onset of ZGA in a well-controlled manner.^23^

Given that the epigenetic changes of chromatin impact regulatory proteins’ accessibility to genomic DNA, PTMs to the core histones are critical for developing both a transcriptionally repressive state for silent genes and a transcriptionally active state for ZGA genes.^106^ Contrary to the histone proteins, the histone-modifying enzymes increase their expression drastically before the major wave of ZGA and reach critical levels, Figure 4B. Among them, Ctr9, Wdr5 and Nsd1b are transcription regulators regarding trimethylation of lysine 4 of histone H3 (H3K4me3), a canonical mark for the transcription start site of active genes.^107^ The expression pattern of these three enzymes is consistent with the previous ChIP-chip profiles and the western blot result of H3K4me3.^108^ Carm1, which specifically catalyzes H3R26me2, was reported to participate in blastocyst development of porcine embryos.^109^ Mier1 functions as a transcriptional repressor by recruitment of Hdac1 and has a similar expression change as Mta2, demonstrating that histone deacetylases (HDACs) are indispensable for ZGA by creating correct transcriptionally repressive and active states.^110^ The functions of Mier1 and Nsd1b in regulating the MZT of zebrafish embryos are still not clear. Our proteomics dataset documents the expression profiles of multiple critical enzymes related to histone modifications, providing some clues about their potential biological functions in transcription regulation during the MZT of zebrafish embryos.

#### Maternal mitoribosomes for energy conversion before MBT

Ribosomal proteins play an essential role in ribosome assembly and protein translation and are also required to form tissues or organs.^111^ As shown in Figure S5A, 20 of the ribosomal proteins and 13 of the mitochondrial ribosomal proteins showed significant abundance changes during MZT. Interestingly, unlike the ribosome proteins, several mitoribosomal proteins, such as Mrps27 and Mrpl53, are highly expressed before MBT (∼3 hpf), implying their function in synthesizing proteins for energy conversion before MBT. More studies are certainly needed to understand the connections between the expression dynamics of ribosomal proteins and the MZT. Our quantitative proteomics data provides a list of ribosomal proteins for further functional studies, and for the first time, delivers a comprehensive picture of ribosomal protein dynamics during the MZT of zebrafish embryos.

#### RNA helicase

The RNA helicases unwind nucleic acid duplexes and, thus, are required for different cellular processes involving RNA. As shown in Figure 4C, eleven RNA helicases show significant expression changes during the MZT with four downregulated helicases and seven upregulated ones. The downregulated RNA helicases most likely function as repressors of transcription and cellular differentiation during MZT, like Ddx6, which impedes cellular differentiation through the degradation of mRNAs of TFs responsible for differentiation.^89^ There is no clear evidence in the literature about the functions of the other three downregulated helicases (Ddx59, Dhx29, and Ddx61) during the MZT of vertebrate embryos. However, their expression profiles provide some clues about their potential functions in regulating MZT. The seven upregulated RNA helicases including Hells, Ddx39a, Ddx19b, Dhx38, Ddx3, Ddx5, and Chd7, play essential roles in promoting transcription, cellular differentiation, and early organogenesis.^55^^,^^56^^,^^64^^,^^65^^,^^66^^,^^68^^,^^69^^,^^70^^,^^112^

#### Glycolysis-associated proteins

To meet the energy demands of embryonic cells during the MZT as they activate transcription, remodel the cell cycle, and promote cell movement, changes in the glycolytic cycle are needed.^113^ In our work, six glycolytic enzymes are upregulated in different patterns during the MZT, including Eno1, Eno3, Ldha, Ldhb, Pgam1a, and Pgam1b, Figure S5B, suggesting their potential roles in supporting the MZT.

#### P-bodies, proteasome, and protein ubiquitination-related enzymes

The maternal products are eliminated during the process of MZT, coordinated with the initiation of zygotic transcription.^1^ Given that the degradation of mRNAs is initially accomplished by maternally encoded products,^114^ the expression pattern of the proteins involved in degradation was investigated. As shown in Figure 4D, we revealed that the proteins involved in processing bodies (P-bodies),^87^^,^^115^^,^^116^ which function in mRNA degradation, translation repression, and mRNA storage, were most likely significantly downregulated during the MZT, like Ybx1, promoting the maternal mRNA clearance. Almost all the differentially expressed proteasome subunits involved in protein degradation display significant accumulation at the dome stage or after, Figure 4E, indicating substantially higher demand for protein degradation when embryos go through the MZT. On the other hand, the ubiquitin-conjugating enzymes and ligases show more diverse expression patterns, Figure S5C. In total, 16 differentially expressed ubiquitination-related enzymes are identified. Seven of them show a clear trend of downregulation and nine of them show two different waves of upregulation, either significantly up regulated at the dome stage or at the 50%-epiboly. The data here presents a global view of genes involved in the ubiquitin-dependent proteolytic pathway during the MZT of zebrafish embryos.

We performed STRING^105^ protein-protein interaction analysis of the differentially expressed TFs, histones, histone-modifying enzymes, RNA helicases, and P-body proteins, Figure 4F. Those proteins are highly associated with each other physically or functionally with at least medium confidence (≥0.4), especially proteins within each group. Ctcf, as a TF, is critical for connecting those different groups of proteins. Six TFs and one histone-modifying enzyme (Mier1a) have no physical or functional connections with other proteins in the network according to the literature. Our proteomics data show that they are differentially expressed during the MZT together with other TFs and histone-modifying enzymes, implying that they could be involved in the network with either physical interactions or functional connections with other proteins in Figure 4F. The data here also suggests that the MZT is well controlled at multiple levels via collaborations between different regulators.

Overall, our protein expression pattern-based cluster and functional analysis of differentially expressed proteins accurately reflect the major events occurring during the MZT. Maternal mRNAs are selectively cleared during the MZT via, e.g., P-bodies, and maternal proteins are selectively degraded through, e.g., ubiquitin-dependent proteolytic pathway. Minor ZGA happens during the embryonic cleavage period, supported by the detectable protein signal from zygotic mRNAs at the 64-cell stage. Cellular differentiation and early organogenesis are initiated by the accumulation of activators including TFs (i.e., Sall4, Hopx, Homeza, and Mlx), helicases (i.e., Dhx38, Ddx3b, Chd7, and Ddx39aa), and others (i.e., Cdc42). Those processes are also precisely controlled by the downregulation of suppressors, i.e., Ddx6 and Ttf2. ZGA is triggered because of the accumulation of activators, i.e., TFs, Figure 3, the dilution of repressors, i.e., histone variants (Figure 4A), and downregulation of repressors, i.e., Ybx1, Dmap1, and Rad50. Several enzymes related to histone PTMs (i.e., Ctr9, Wdr5 and Nsd1b), Figure 4B, are crucial for epigenetic control of the MZT. More importantly, our work provides a list of potential regulators of MZT in zebrafish embryos, e.g., TFs, histones, histone-modifying enzymes, and RNA-helicases. The data bettered our understanding of the full picture of MZT, which is well controlled at multiple levels in a collaborative manner, Figure 4F. Additionally, the energy requirement is substantially increased during the progression of MZT, which is ensured by an accumulation of mitochondrial proteins (Figure 2A, cluster 1) and glycolytic process-related proteins (Figure S5B).

### Comparison of mRNA and protein expression patterns during MZT of zebrafish embryos

To investigate whether and how changes in the transcriptome lead to proteome changes during the MZT of zebrafish embryos, we integrated our proteomic data with a published transcriptome result,^31^ which has served as a time-resolved transcriptional basis for zebrafish genomic changes and has been a helpful resource in systems biology. We used the transcriptome data at four embryonic stages closest to our proteomic data points during the MZT, including the 128-cell, 1k-cell, dome, and 50% epiboly stages. We noted some differences in the embryonic stage between the transcriptome and proteome datasets. However, here, we focus on the expression profile comparison and the proteome and transcriptome time points are either the same (i.e., dome and 50% epiboly) or close to each other (i.e., 64-cell vs. 128-cell, 256-cell vs. 1k-cell). Therefore, the comparison should provide reliable messages about the discrepancy and consistency between mRNA and protein dynamics during the MZT. The protein-mRNA pairs are listed in Table S6.

The distribution of expression levels (TPM, transcripts per million) were applied for temporal expression analysis of mRNA data using the program maSigPro^48^ with a degree = 3 (no. of time points −1). Transcripts with absolute log_2_ fold change larger than 1 between any two time points and with an adjusted FDR corrected *p*-value<0.05, were determined as mRNAs with statistically significant abundance changes.

**Figure 5:**
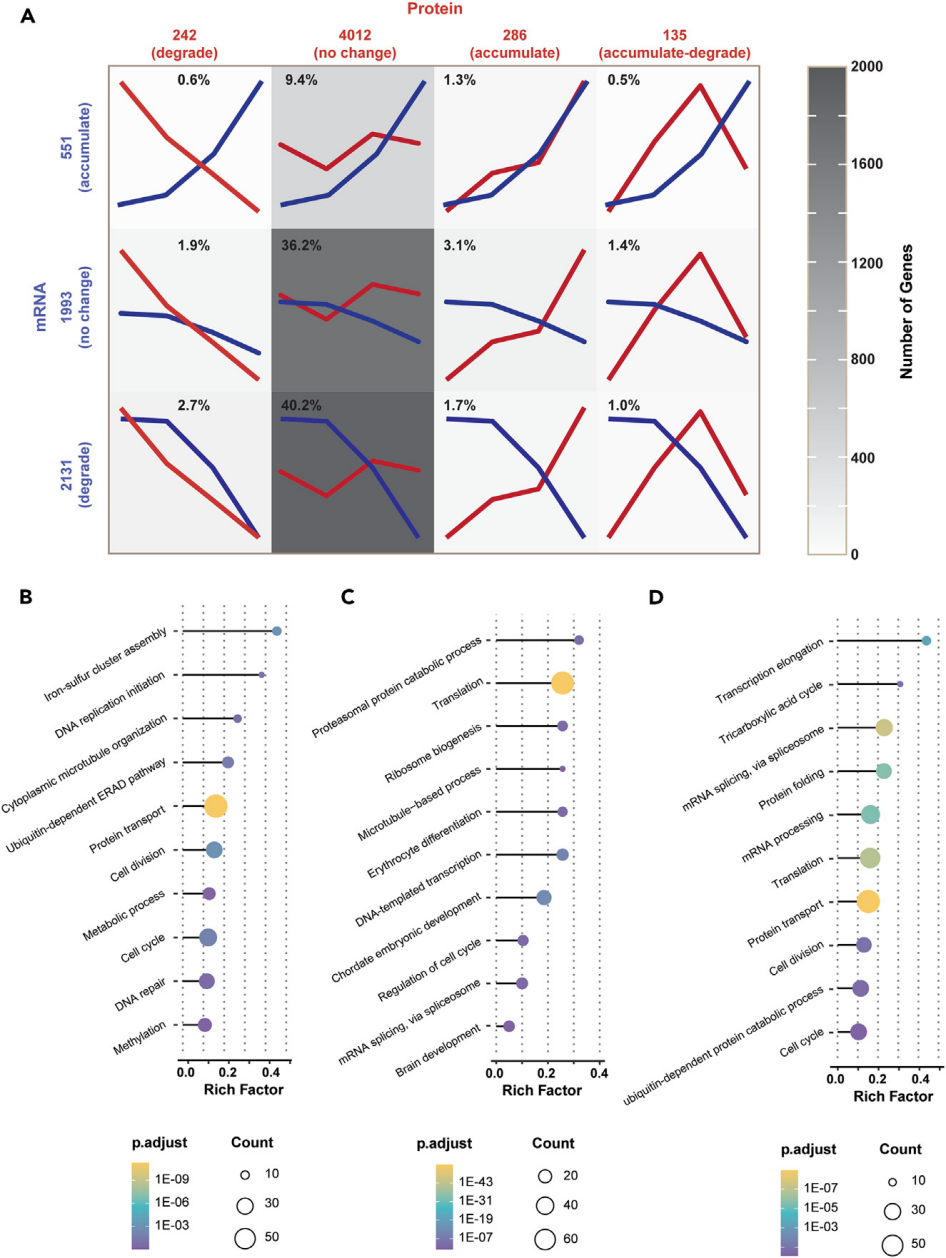
Discordance of temporal patterns in zebrafish transcript and protein expression during the MZT (A) Mutual information between the temporal pattern of mRNA and protein expression presented as co-clustering into three main trends (mRNA) and four main trends (protein). The grayscale background reflects the number of genes in each group. Only proteins or mRNAs that show significant fold changes with *p*-values ≤0.05 were considered as differentially expressed ones for the co-clustering analysis. The four time points covered by the proteome data are 64-cell, 256-cell, dome, and 50% epiboly stages. The time points covered by the transcriptome data are 128-cell, 1k-cell, dome, and 50% epiboly stages. Top 10 enriched biological processes of genes corresponding to the degraded mRNAs (B), the accumulated mRNAs (C), and the no-change mRNAs (D). The size of circle represents gene count involved in each biological process. The enriched biological processes are colored by adjusted *p*-value. The smaller the *p*-value is, the more significant enrichment of the particular biological process associated with the group of genes is.

To elucidate mRNA-protein dynamics, we classified the temporal behavior (accumulate, no change, degrade, and accumulate followed by degrade) of the 4675 mRNA-protein pairs across the four development stages with qualitatively distinct dynamics using k-means clustering approach, Figure 5A. We have several main observations. First, abundance of mRNAs is drastically more dynamic than proteins. About 57% of the mRNAs have statistically significant changes in abundance and only 14% of proteins have significant expression changes across the studied embryonic stages. The phenomenon is most likely due to several reasons. Maternal mRNAs are gradually cleared during the MZT, as evidenced that about 46% of the mRNAs started to be degraded significantly after the MBT. Zygotic mRNAs increase gradually after the MBT, as evidenced by the significant accumulation of 551 mRNAs after the MBT. In addition, the poly(A) tail length of mRNAs increases when zebrafish embryos develop from 2 hpf to 6 hpf during the MZT.^117^ The poly(A) tail lengthening could benefit the RNA-seq measurement by White et al.,^31^ leading to increase of mRNA abundance as zebrafish embryos develop through the MZT, which makes the interpretation of the mRNA expression patterns more complicated. For example, the slight abundance increases of the 551 “accumulate” mRNAs from 128-cell to 1k-cell stage might be simply due to the poly(A) tail lengthening instead of minor ZGA. Second, the transcriptome profiles accurately reflect the main events that happened during the MZT. Over 2100 maternal mRNAs are gradually degraded after the MBT, ensuring the onset of ZGA. Those genes are highly enriched in cell division, cell cycle, ubiquitin-dependent ERAD pathway, DNA replication, and methylation (i.e., DNA, RNA, and histone), Figure 5B, agreeing with the lengthening DNA replication and cell cycle, protein degradation, and multi-level regulation of ZGA in zebrafish embryos. About 550 mRNAs are accumulated to coordinate with the ZGA and early organogenesis. Those genes are highly enriched in transcription, mRNA splicing, translation, regulation of cell cycle, chordate embryonic development, erythrocyte differentiation, and brain development, Figure 5C. Over 1990 mRNAs have no abundance change and are highly enriched in transcription elongation, mRNA splicing, mRNA processing, translation, protein folding, protein transport, cell cycle, cell division, and tricarboxylic acid cycle, Figure 5D. Third, the proteome and transcriptome profiles have drastic discrepancy during the MZT. Only 41% of protein-mRNA pairs show consistent expression profiles. About 36% of them show no significant changes at both protein and mRNA levels; about 5% of them are differentially expressed and have a reasonable agreement between protein and mRNA, i.e., Ctcf, Sall4, Ddx6, and Eif4enif1. About 59% of protein-mRNA pairs show significant disagreement between protein and mRNA profiles, Figure 5A. For example, 50% of the pairs have no expression change at the protein level but are substantially up- (9.4%) or downregulated (40.2%) at the mRNA level. We noted that the poly(A) tail lengthening during the MZT^117^ may contribute to the disagreement between mRNAs and proteins for the 9.4% upregulated mRNAs. About 6.4% of the pairs having no statistically significant expression change at the mRNA level are differentially expressed at the protein level (1.9%, downregulated; 3.1% upregulated; 1.4% upregulated and then downregulated). Very interestingly, about 2.3% of the pairs have completely opposite expression profiles at the protein and mRNA levels (1.7% mRNA downregulated and protein upregulated; 0.6% mRNA upregulated and protein downregulated), including crucial TFs for modulating the MZT, i.e., Nanog and Ybx1.

There are several potential reasons for the substantial expression profiles discrepancies in proteome and transcriptome during the MZT. First, maternal mRNAs are directly deposited into embryos and there is minimal transcription before the major ZGA. Post-transcriptional regulations need to take place to ensure the accurate control of protein expression upon request. Second, the translation process needs time, so proteins are expected to be synthesized and accumulated after a delay relative to mRNA. Third, mRNAs can be packaged in granules, such as P-bodies. Once these mRNAs are released, they will be immediately available for translation. Meanwhile, the maternal mRNAs are subjected to degradation, leading to potential upregulation of proteins and downregulation of mRNAs. Fourth, the protein synthesis and degradation rates determine the overall expression profile, resulting in another possible factor for the disagreement between protein and mRNA profiles. The discrepancy between proteome and transcriptome profiles of zebrafish early-stage embryos agrees well with the data of *Xenopus* embryos.^118^ The discrepancy also demonstrates that mRNA may not serve as a reliable proxy for protein expression during the MZT, implying the fundamental importance of studying the proteome to decipher the molecular mechanisms of the MZT.

### Limitations of the study

The current study still has three limitations. First, our proteome coverage is still limited due to the interference of high-abundance yolk proteins. We expect further improving the peptide separations and using a high-end mass spectrometer with much faster scan rates and higher sensitivity will help to reach a deeper proteome coverage. Second, the number of embryonic stages covered in this study is limited to four during the MZT, which impedes our global view of zebrafish proteome dynamics with high time resolution during the early embryogenesis. Third, due to the limitations of bottom-up proteomics, our proteome dataset cannot offer expression dynamics of specific proteoforms, which arise from the same gene due to gene-level variations, RNA-level alternative splicing, and protein PTMs.^119^^,^^120^ Proteoforms from the same gene usually have similar sequences. We expect that application of MS-based top-down proteomics^120^ to study early embryogenesis will substantially advance our understanding of protein function in modulating the important events during, e.g., MZT.

## STAR★Methods

### Key resources table

**Table:** 

REAGENT or RESOURCE	SOURCE	IDENTIFIER
**Biological samples**		

Zebrafish	ZFIN The Zebrafish Information Network	ZDB-GENO-150204-3

**Chemicals, peptides, and recombinant proteins**		

Mammalian cell-PE LB™ cell lysis	G-Biosciences	Cat #786-180
cOmplete™ ULTRA Tablets, Mini, EASYpack Protease Inhibitor Cocktail	Roche	Cat #05892970001
PhosSTOP™	Roche	Cat #4906845001
Acetone	Fisher Scientific	Cat #A949-1
Sodium dodecyl sulfate (SDS)	Sigma-Aldrich	Cat #L6026-50G
Ammonium bicarbonate (NH_4_HCO_3_)	Sigma-Aldrich	Cat #09830-500G
Water	Fisher Scientific	Cat #W64
DL-Dithiothreitol (DTT)	Sigma-Aldrich	Cat #43815-1G
Iodoacetamide (IAA)	Sigma-Aldrich	Cat #I1149-5G
Urea	Thermo Fisher Scientific	Cat #J65769.36
Trypsin from bovine pancreas	Sigma-Aldrich	Cat #T1426-1G
Formic Acid (FA)	Fisher Scientific	Cat #A117-50
Triethylammonium bicarbonate buffer	Sigma-Aldrich	Cat #T7408-100ML
Trizma® hydrochloride (Tris-HCl)	Sigma-Aldrich	Cat #T3253-500G
Acetonitrile (ACN)	Fisher Scientific	Cat #A955-4
Acetic Acid (AA)	Fisher Scientific	Cat #A1131AMP
Methanol	Fisher Scientific	Cat #A456-4

**Critical commercial assays**		

BCA Protein Assay Kit	Thermo Fisher Scientific	Cat #23227
Microcon® Centrifugal Filters	Sigma-Aldrich	Cat #MRCF0R030
Sep-Pak C18 1 cc Vac Cartridge	Waters	Cat #WAT054955
iTRAQ Reagent-8Plex One Assay Kit	Sciex	Cat #4390811

**Deposited data**		

Mass spectrometry data	This paper	ProteomeXchange: PXD036678

**Software and algorithms**		

MaxQuant (v1.5.5.1)	Cox Lab	https://maxquant.org/
R (v4.2)	R Project	https://www.r-project.org
RStudio	Open source	https://www.rstudio.com/
Perseus (v1.6.15.0)	Cox Lab	https://maxquant.net/perseus/
UniProt	UniProt Consortium	https://www.uniprot.org/
maSigPro (v1.74)	Bioconductor	https://bioconductor.org/packages/release/bioc/html/maSigPro.html
ClusterGVis	junjunlab	https://github.com/junjunlab/ClusterGVis
ggplot2	CRAN	https://cran.r-project.org/web/packages/ggplot2/index.html
DAVID	Open source	https://david.ncifcrf.gov/
String	N/A	https://string-db.org
Cytoscape 3.7.2	Open source	https://cytoscape.org

### Resource availability

#### Lead contact

Further information and requests for resources should be directed to and will be fulfilled by the lead contact, Liangliang Sun (lsun@chemistry.msu.edu).

#### Materials availability

This study did not generate new unique reagents.

#### Data and code availability

• The MS raw data has been deposited to the ProteomeXchange Consortium via the PRIDE^121^ partner repository and can be publicly accessible with the dataset identifier PXD036678. We have built a website (https://www.toppic.org/software/zebrafishdb/index.html) to facilitate developmental biologists to get access to the quantitative proteomics dataset.
• This paper does not report the original code.
• Any additional information required to reanalyze the data reported in this paper is available from the lead contact upon request.

### Experimental model and study participant details

#### Zebrafish maintenance and breeding

Zebrafish (NHGRI-1 strain) are housed in a ZMod self-enclosed system, with all procedures following ZIRC/AALAC guidelines (https://www.aaalac.org/pub/?id=E9019693-90EC-FC4A-526E-E8236CC13B28). Water temperature was set at 28.5°C and pH at 7.2–7.4, with pH, ammonia, and nitrites checked daily. In addition, 25% of the water in the system was changed daily. Each holding tank has 2-L water capacity and has a maximum number of 12 adult fish per tank. Tanks are made of polypropylene, and fish are moved to a clean tank approximately every 4 weeks. Fish were on a 14h light/10h dark-light cycle. One day before the breeding day, a male and a female fish were placed in the breeding tank with a sieve separator. On the breeding day, soon after the lights came on, the separator was removed, and fish were briefly allowed to breed naturally. After the first indication that natural breeding was about to occur, the fish were separated so the sperm and eggs could be manually collected for *in vitro* fertilization (IVF). With the sperm and eggs collected, the male and female fish were placed back into tanks and rested for at least one month before the next round of breeding or experimental handling. The maintenance and breeding of zebrafish were performed by the Cibelli group in the Department of Animal Science at Michigan State University. The whole protocol was operated in compliance with national and institutional guidelines for animal research.

#### Embryo collection

For the embryo development analysis experiment, 30 IVF-produced embryos at each of 4 different early stages (64-cells, 256-cells, dome, and 50%-epiboly) were collected and split into two 1.7 mL centrifuge tubes (15 embryos/tube). The redundant liquid was carefully removed with a 200-μL pipette. The tubes were immediately cooled down by liquid nitrogen and stored at −80°C before use.

### Method details

#### Sample preparation for MS analysis

The embryos collected at four stages in each tube were suspended in 700 μL of mammalian cell-PE LB cell lysis buffer containing complete protease inhibitor and phosphatase inhibitor. After homogenization for 1 min on ice, all the tubes were sonicated for 10 min on ice using a Branson Sonifier 250 (VWR Scientific, Batavia, IL). The lysates were then centrifuged at 12,000 g for 5 min. The supernatants were collected, and a small portion was used to measure the protein concentration with a BCA assay. Based on the measured concentration, 200 μg of protein sample at each stage were purified by acetone precipitation: 1 volume of protein sample was mixed with 4 volumes of cold acetone, and the mixtures were kept at −20°C overnight. The tubes were then centrifuged at 10,000 g for 5 min, and the supernatants were discarded. The pellets were simply washed using 500 μL of cold acetone and re-centrifuged. The supernatants were discarded, and the protein pellets were placed in a chemical hood for 1–2 min, leaving ∼5 μL of liquid. The protein samples were stored at −80°C before use. All the protein pellets were suspended in 100 μL of 2% SDS (w/v) and 100 mM NH_4_HCO_3_ solution, vortexed, and sonicated. To denature the proteins, tubes were kept at 90°C for 20 min. To reduce the disulfide bonds between cysteine amino acid residues, 6 μL of 0.1 M of DTT were added to each tube and tubes were kept at 80°C for 20 min. Protein alkylation with 15 μL of 0.1 M IAA was done at room temperature in the dark for 20 min 6 μL of 0.1 M DTT was added to each tube to react with the residue IAA. The sample was then mixed with 125 μL 8 M urea in 100 mM NH_4_HCO_3_, followed by FASP sample preparation^122^ with minor modification. Then the mixture of each tube was loaded onto a 30-kDa centrifugal filter unit (250 μL/unit) followed by centrifugation at 12,000 g for 10 min. The proteins on the membrane were washed with 250 μL of 8 M urea in 100 mM NH_4_HCO_3_ three times. Next, the proteins were washed with 100 mM NH_4_HCO_3_ three times to remove urea. Finally, 150 μL of 100 mM NH_4_HCO_3_ (pH 8.0) was loaded on each membrane and 7 μL of trypsin solution (1 μg/μL) was added to each unit. The filter units were gently vortexed for 5 min to mix the trypsin and proteins. After that, the filter units were kept in a 37°C water bath overnight for tryptic digestion. After digestion, the units were centrifuged at 12,000 g for 15 min, and the flow-through containing the peptides was collected. To increase peptide recovery from the membrane, the membrane was further washed with 150 μL of 100 mM NH_4_HCO_3_. FA was used to acidify the protein digests to terminate digestion (0.5% FA final), followed by desalting using C18 SPE columns (50 mg beads, 250 μg peptide capacity) (Waters, Milford, MA); the elutes from desalting were lyophilized with a vacuum concentrator (Thermo Fisher Scientific). The digests were stored at −80°C before use.

#### iTRAQ labeling followed by high-pH RPLC fractionation

For the samples collected at four stages for protein profiling, two biological replicates at each time point were included. The peptides from the total of eight samples were labeled with iTRAQ 8-plex reagent by following the manufacturer’s instruction (Figure 1A). Briefly, the lyophilized digests were dissolved in 70 μL of 500 mM Triethylammonium bicarbonate (TEAB) buffer (Dilute the 1 M buffer with water, make sure the pH > 7.5). We then withdrew 35 μL of the solution and added 50 μL of isopropanol to each iTRAQ reagent vial. The iTRAQ reagent was transferred to each sample and incubated for 2 h. Next, 50 μL of 100 mM Tris-HCl (pH 8.0) buffer was added to each tube, followed by incubation at room temperature for 40 min to block the residue iTRAQ reagent. Then the digests were mixed and acidified by FA to 0.5% FA final concentration. The sample was lyophilized to remove the organic solvent. When there was ∼200 μL solution left, the lyophilization was stopped. 600 μL of 0.5% FA was added to the sample (total volume 800 μL). The samples were desalted with two C18 SPE columns (100 mg beads, Waters, Milford, MA) and lyophilized. The sample was re-dissolved in 0.1% FA, 2% ACN (800 μL) and kept at −20°C before use.

An Agilent Infinity II HPLC system was used for high pH RPLC fractionation. A Zorbax 300Extend-C18 RP column (2.1 mm i.d. × 150 mm length, 3.5 μm particles, Agilent Technologies) was used for separation. Mobile phase A was 5 mM NH_4_HCO_3_ in water with pH 9 and mobile phase B was 5 mM NH_4_HCO_3_ in 80% ACN with pH 9. Gradient elution was used for peptide separation. 800-μg iTRAQ-labeled zebrafish embryo digest was injected onto the RP column. The flow rate was 0.3 mL/min. The gradient was as follows: 0–5 min, 2% B; 5–8 min, 2–10% B; 8–108 min, 10–50% B; 108–110 min, 50–100%B; 110–120 min, 100% B; 120–122 min, 100-2% B; 122–132 min, 2% B. In total, 100 fractions were collected from 8 min to 108 min, one fraction per min. We named the fractions based on the order of retention time from 1 to 100. Then we combined fraction N and fraction N+50 to generate 50 fractions. Those fractions were then lyophilized and stored at −80°C for low pH RPLC-MS/MS and CZE-MS/MS analysis.

#### RPLC-MS/MS for the iTRAQ-labeled sample

An EASY-RPLC 1200 system (Thermo Fisher Scientific) was used for RPLC separation. Each of the 50 high-pH RPLC fractions was dissolved in 10 μL of 0.1% (v/v) FA and 2% (v/v) ACN. 2 μL of the sample was injected onto a C18 pre-column (Acclaim PrepMapTM 100, 75-μm i.d. × 2 cm, nanoviper, 3 μm particles, 100 Å, Thermo Scientific). Then, the loaded peptides were separated on a C18 separation column (Acclaim PrepMapTM 100, 75-μm i.d. × 50 cm, nanoviper, 2 μm particles, 100 Å, Thermo Scientific) at a flow rate of 200 nL/min. Mobile phase A was 2% (v/v) ACN in water containing 0.1% (v/v) FA), and mobile phase B was 80% (v/v) ACN and 0.1% (v/v) FA. For separation, a 150-min gradient was used: 0–80 min, 8–30% B; 80–120 min, 30–55% B; 120–135 min, 55–100% B, 135–150 min, 100% B. The LC system required another 30 min for column equilibration between runs. Therefore, one LC-MS run required about 2 h. A Q-Exactive HF mass spectrometer (Thermo Fisher Scientific) was used for the RPLC-MS/MS experiments. The spray voltage was set to 1.8 kV. A Top10 DDA method was used. The mass resolution was set to 60,000 (at m/z 200) for both full MS scans and MS/MS scans. For full MS scans and MS/MS scans, the target value was 3E6 and 1E5, and the maximum injection time was 50 ms and 110 ms, respectively. The scan range for MS scans was 300–1500 m/z. For MS/MS scans, the isolation window was 2 m/z. Fragmentation in the HCD cell was performed with a normalized collision energy of 28%. The fixed first mass was set to 100 m/z. Dynamic exclusion was applied and it was set to 30 s. Ions with unassigned or +1 charge states were not considered for fragmentation.

#### CZE-MS/MS for the iTRAQ-labeled sample

An ECE-001 capillary electrophoresis autosampler (CMP Scientific, Brooklyn, NY) was used for CZE separation. A commercialized electro-kinetically pumped sheath flow interface (CMP Scientific, Brooklyn, NY)^123^ was employed for coupling CZE to MS. A 1-m-long LPA coated capillary (50 μm i.d., 360 μm o.d.) made in house^124^ was used for CZE. A Sutter instrument P-1000 flaming/brown micropipette puller was used to pull a borosilicate glass capillary (1.0 mm o.d., 0.75 mm i.d., and 10 cm length). The resultant electrospray emitter has an opening with a diameter of 30–40 μm. The background electrolyte (BGE) of CZE was 5% (v/v) acetic acid (pH 2.4) and the sheath buffer was 0.2% (v/v) FA containing 10% (v/v) methanol. 4 μL of leftover iTRAQ-labeled samples in RPLC-MS/MS analysis was withdrawn and lyophilized. The dry sample was re-dissolved in 3 μL of 50 mM NH_4_HCO_3_ (pH 8.0). The sample was injected using 5-psi pressure for 95 s. 30 kV was applied at the injection end for CZE separation, and around 2 kV was applied in the sheath buffer vial for electrospray. For all the CZE–MS runs, the separation was performed for 90 min, followed by 10 min wash BGE for 10 min at 5 psi pressure.

#### Live imaging

Zebrafish embryos were imaged using an Olympus IX73 microscope with an Olympus DP23 camera and cellSens Entry software.

### Quantification and statistical analysis

#### Database search

The raw files for CZE-MS/MS and RPLC-MS/MS were respectively analyzed by MaxQuant 1.5.5.1 software with the Andromeda search engine.^125^ For the iTRAQ-labeled datasets from zebrafish, iTRAQ on lysine and N-terminus was selected as peptide labels. The *Danio rerio* proteome (ID: UP000000437, 46,848 entries, Oct 12, 2021) downloaded from UniProt (http://www.uniprot.org/) was used for database search. The peptide mass tolerances of the first and main searches were 20 and 4.5 ppm, respectively. The fragment ion mass tolerance was 20 ppm. Trypsin was selected as the protease. The dynamic modification was oxidation on methionine and acetylation on protein N-terminus, and carbamidomethyl on cysteine was set as static modifications. The minimum length of a peptide was set to 7. The FDRs were 1% for both peptide and protein.

#### Statistical analysis

For the time-series experiment, the statistical analysis was performed using the statistical software R (R version 4.2). Firstly, the database search results were respectively subjected to Perseus software (v1.6.15.0).^126^ All entries marked by MaxQuant as a "Potential contaminant", "Reverse" or "Only identified by site" were deleted, followed by the removal of the protein containing intensity as zero. To address heterogeneity of variance, the variance-stabilizing normalization (VSN) transformation was respectively performed to the reporter ion intensities from CZE-MS/MS and RPLC-MS/MS methods with Normalyzer.^127^ After normalization, the expression level of all quantified proteins in 8 channels (biological replicates of zebrafish embryos at 64-cell, 256-cell, dome and 50%-epiboly stage) was respectively divided by the data in the first channel (64-cell-stage). For the proteins commonly quantified in both RPLC-MS/MS and CZE-MS/MS method, the ratio in each channel of two methods were respectively averaged. For those exclusively quantified with RPLC-MS/MS or CZE-MS/MS method, keep the origin ratio value. Then, the normalized read counts were applied for temporal expression analysis using the program maSigPro^48^ with a degree = 3 (no. of time points −1). Finally, mfuzz model was employed to cluster together significant genes with similar expression patterns and visualized using the ClusterGVis package.^49^

For the time-series transcriptome data from published work,^31^ the distribution of expression levels (TPM, transcripts per million) were applied for temporal expression analysis using the program maSigPro^48^ with a degree = 3 (no. of time points −1). Transcripts with absolute log2 fold change>1 between any two time points, with an adjusted FDR corrected *p*-value<0.05, were determined as mRNAs with statistically significant abundance changes. Then, the obtained proteins and mRNAs with statistically significant abundance changes were respectively clustered into 4 or 3 clusters using k-means clustering approach and visualized with ggplot2 package.

#### Gene ontology and protein-protein interaction network

For the gene ontology (GO) analysis, the web-based DAVID Bioinformatics Resources (https://david.ncifcrf.gov/) was employed with the default settings.^128^^,^^129^

The protein-protein interaction network analyses were done with the STRING database (https://string-db.org/).^105^ Network type is full STRING network and the edges indicate both physical and functional associations of proteins. The network edge suggests the confidence of protein-protein connection and the line thickness indicates the strength of data support with thicker lines as more confident connections. The minimum required interaction score is medium confidence (0.4). Others are default settings and visualized using the Cytoscape software.^130^

## Supplemental information

Document S1. Figures S1–S5

Table S1. Proteins identified by RPLC-MS/MS and CZE-MS/MS methods from zebrafish embryos, related to Figure 1

Table S2. Expression pattern of all proteins quantified by RPLC-MS/MS and CZE-MS/MS from zebrafish embryos, related to Figure 2

Table S3. Expression pattern cluster analysis of differentially expressed proteins quantified by RPLC-MS/MS and CZE-MS/MS from zebrafish embryos, related to Figure 2

Table S4. Functional annotation analysis of differentially expressed proteins from zebrafish embryos in six clusters categorized based on the expression pattern, related to Figure 2

Table S5. Total transcription factors in zebrafish embryo dataset predicted with AnimalTFDB (http://bioinfo.life.hust.edu.cn/AnimalTFDB/#!/), related to Figure 3

Table S6. Proteins commonly quantified in our proteome dataset and published transcriptome dataset, related to Figure 5
