# Supplementary material for: Quantitative proteomics reveals the dynamic proteome landscape of zebrafish embryos during the maternal-to-zygotic transition
Source: iScience. 2024 May 8;27(6):109944. doi: 10.1016/j.isci.2024.109944 (PMC11111832; doi:10.1016/j.isci.2024.109944)
Supplement: Document S1. Figures S1–S5 [file mmc1.pdf]

iScience, Volume *27*

Supplemental information

Quantitative proteomics reveals the dynamic proteome landscape of zebrafish embryos during the maternal-to-zygotic transition

Fei Fang, Daoyang Chen, Abdul Rehman Basharat, William Poulos, Qianyi Wang, Jose B. Cibelli, Xiaowen Liu, and Liangliang Sun

Supplemental Figures

Figure S1 Physicochemical properties distribution of quantified peptides and proteins, related to Figure 1.

Figure S2 Comparison of the data by our study and the study from Yan et al. [S1], related to Figure 1.

Figure S3 Correlation between two data sets of proteins identified from this work and Yan's work [S1], related to Figure 1.

Figure S4 Correlation between two data sets of differentially expressed proteins identified from this work and Yan's work [S1], related to Figure 1.

Figure S5 Expression patterns of potential regulators beyond TFs that show significant abundance changes during the MZT, related Figure 2.

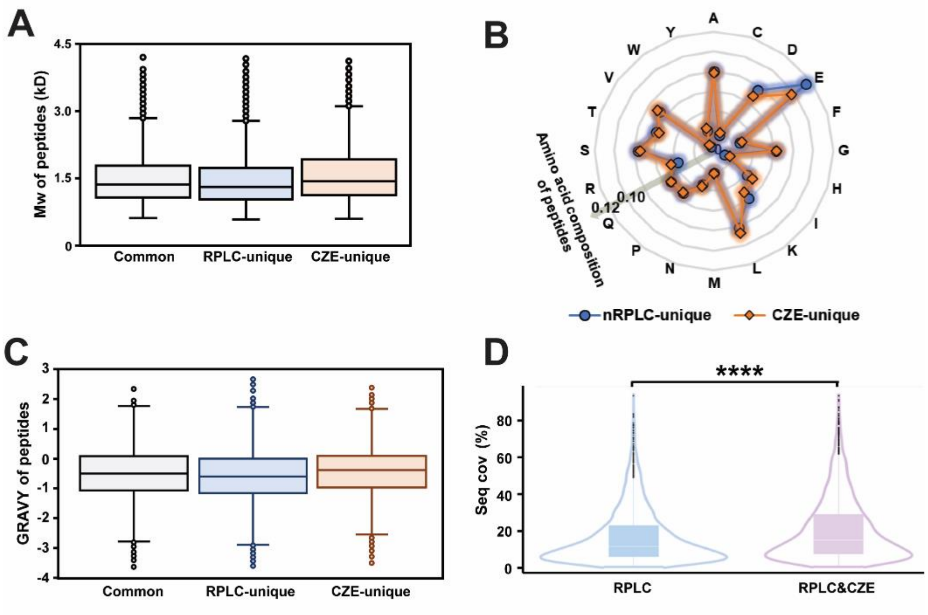

Figure S1 Physicochemical properties distribution of quantified peptides and proteins, related to Figure 1.

(A) Molecular weight (Mw) distributions of peptides commonly identified by RPLC-MS/MS and CZE-MS/MS, uniquely identified by RPLC-MS/MS or CZE-MS/MS. (B) Amino acid distribution of the uniquely identified peptides by RPLC-MS/MS or CZE-MS/MS. (C) GRAVY (Grand average of hydropathicity index) value distribution of the peptides commonly identified by both RPLC-MS/MS and CZE-MS/MS analysis, uniquely identified by RPLC-MS/MS or CZE-MS/MS analysis. Positive GRAVY values represent hydrophobic and negative values indicate hydrophilic. (D) Sequence coverage distribution of proteins identified by RPLC-MS/MS alone and the combination of CZE-MS/MS and RPLC-MS/MS, ****$p<0.0001$, two-tailed, paired t test.

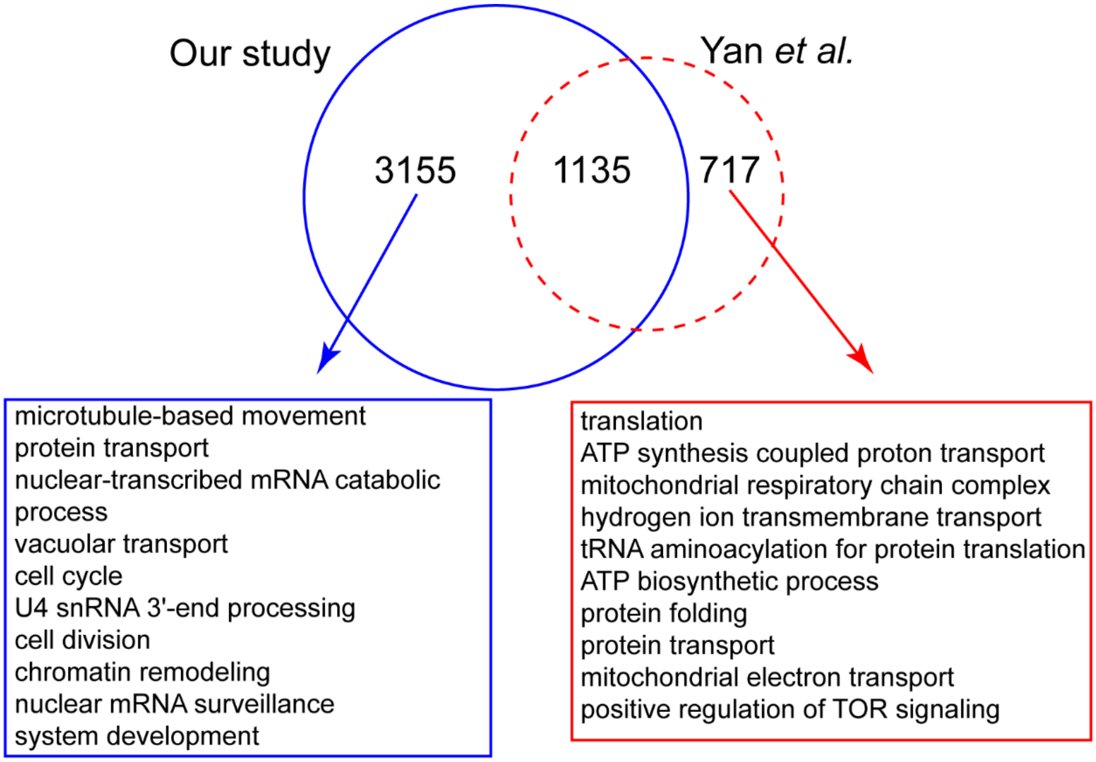

Figure S2 Comparison of the data by our study and the study from Yan et al. [S1], related to Figure 1.

Overlap of genes covered by our study and the study from Yan et al. [S1] at the 256-cell and dome stages. The top 10 enriched biological processes of proteins unique to our study and Yan's work are listed. The DAVID functional annotation bioinformatics microarray analysis (https://david.ncifcrf.gov/) was used to perform the biological process enrichment analysis with a p-value better than 0.0001.

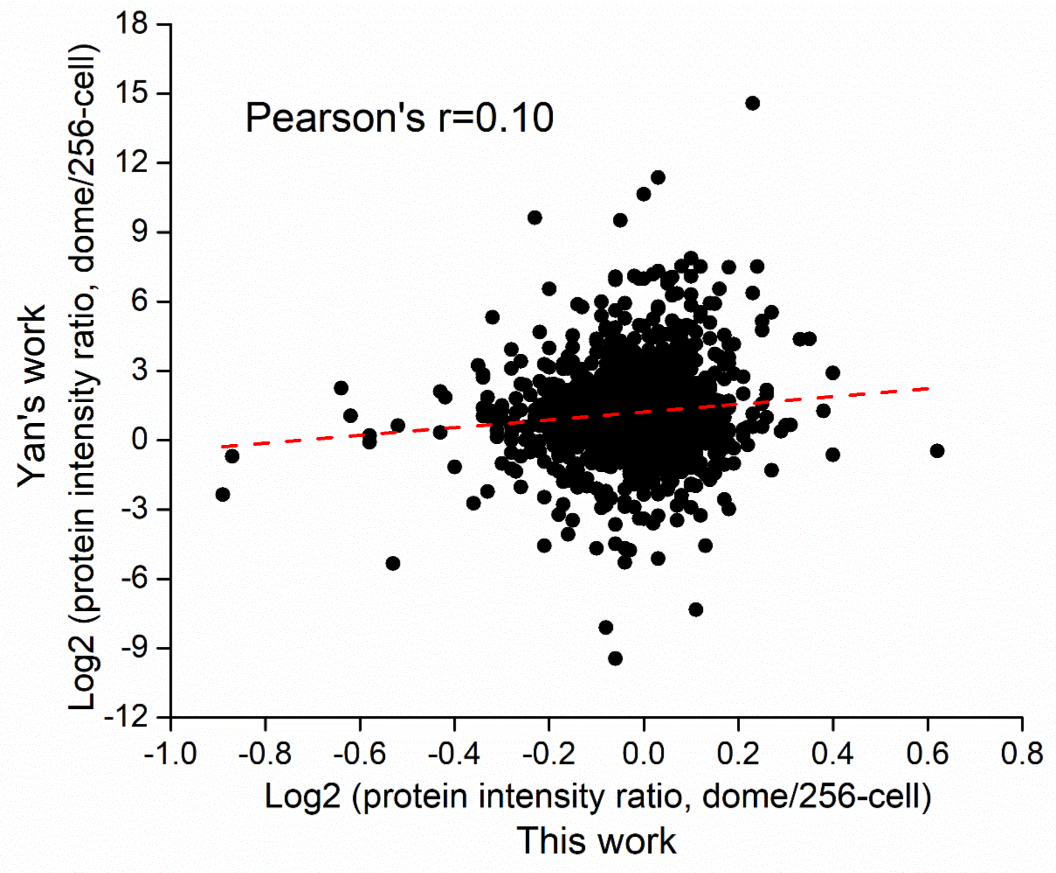

Figure S3 Correlation between two data sets of proteins identified from this work and Yan's work [S1], related to Figure 1.

Linear correlation between this work and Yan's work [S1] in terms of protein intensity ratio between dome and 256-cell stages.

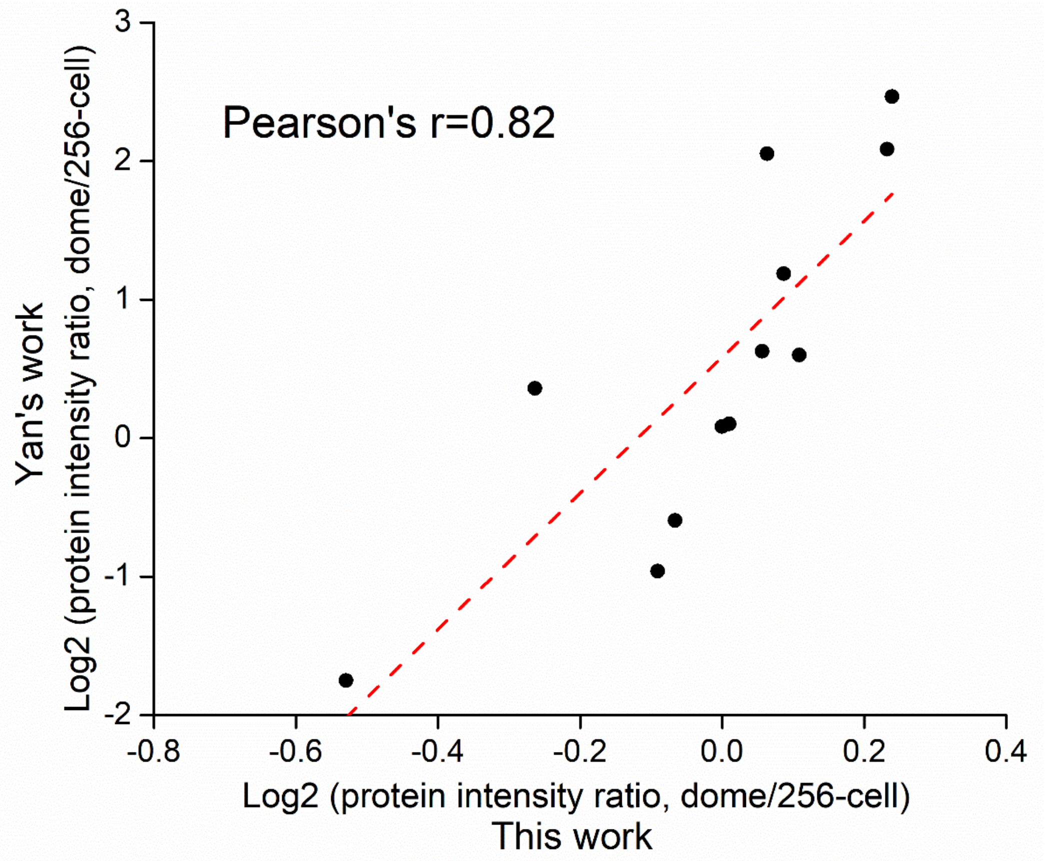

Figure S4 Correlation between two data sets of differentially expressed proteins identified from this work and Yan's work [S1], related to Figure 1.

Linear correlation between this work and Yan's work regarding intensity ratio of differentially expressed proteins during the MZT between dome and 256-cell stages.

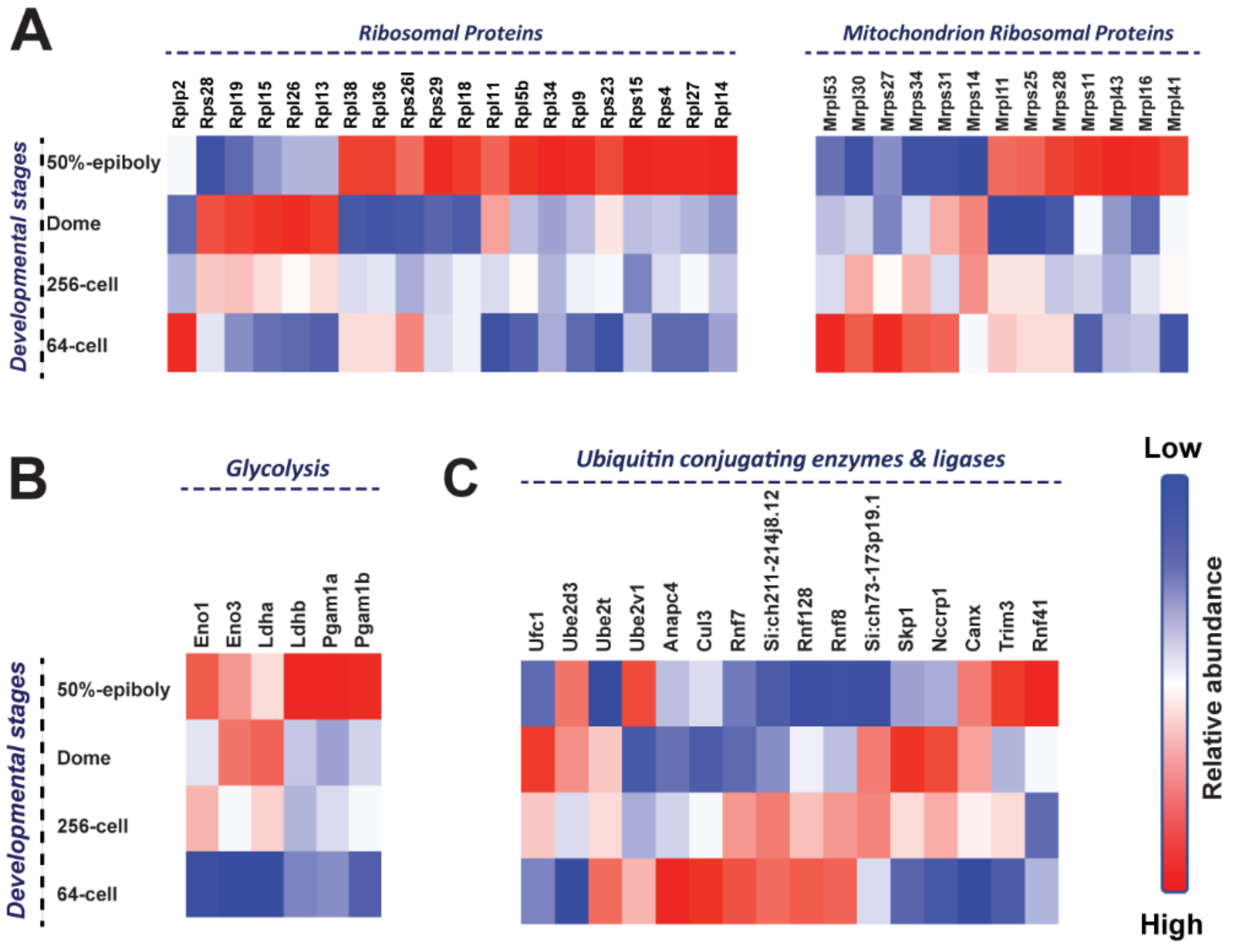

Figure S5 Expression patterns of potential regulators beyond TFs that show significant abundance changes during the MZT, related Figure 2.

Expression patterns of (A) ribosome proteins, (B) glycolysis-associated proteins, and (C) ubiquitin conjugating enzymes and ligases.

References

S1. Yan, J., Ding, Y., Peng, Z., Qin, L., Gu, J., and Wan, C. (2023). Systematic Proteomics Study on the Embryonic Development of Danio rerio. J. Proteome Res. 22, 2814–2826. 10.1021/acs.jproteome.3c00056.
